# An Automated Treatment Plan Quality Control Tool for Intensity-Modulated Radiation Therapy Using a Voxel-Weighting Factor-Based Re-Optimization Algorithm

**DOI:** 10.1371/journal.pone.0149273

**Published:** 2016-03-01

**Authors:** Ting Song, Nan Li, Masoud Zarepisheh, Yongbao Li, Quentin Gautier, Linghong Zhou, Loren Mell, Steve Jiang, Laura Cerviño

**Affiliations:** 1 Department of Biomedical Engineering, Southern Medical University, Guangzhou, Guangdong, China; 2 Center for Advanced Radiotherapy Technologies and Department of Radiation Oncology, University of California San Diego, La Jolla, United States of America; 3 Radiation Oncology Department, University of Texas Southwestern Medical Center, Dallas, Texas, United States of America; North Shore Long Island Jewish Health System, UNITED STATES

## Abstract

Intensity-modulated radiation therapy (IMRT) currently plays an important role in radiotherapy, but its treatment plan quality can vary significantly among institutions and planners. Treatment plan quality control (QC) is a necessary component for individual clinics to ensure that patients receive treatments with high therapeutic gain ratios. The voxel-weighting factor-based plan re-optimization mechanism has been proved able to explore a larger Pareto surface (solution domain) and therefore increase the possibility of finding an optimal treatment plan. In this study, we incorporated additional modules into an in-house developed voxel weighting factor-based re-optimization algorithm, which was enhanced as a highly automated and accurate IMRT plan QC tool (TPS-QC tool). After importing an under-assessment plan, the TPS-QC tool was able to generate a QC report within 2 minutes. This QC report contains the plan quality determination as well as information supporting the determination. Finally, the IMRT plan quality can be controlled by approving quality-passed plans and replacing quality-failed plans using the TPS-QC tool. The feasibility and accuracy of the proposed TPS-QC tool were evaluated using 25 clinically approved cervical cancer patient IMRT plans and 5 manually created poor-quality IMRT plans. The results showed high consistency between the QC report quality determinations and the actual plan quality. In the 25 clinically approved cases that the TPS-QC tool identified as passed, a greater difference could be observed for dosimetric endpoints for organs at risk (OAR) than for planning target volume (PTV), implying that better dose sparing could be achieved in OAR than in PTV. In addition, the dose-volume histogram (DVH) curves of the TPS-QC tool re-optimized plans satisfied the dosimetric criteria more frequently than did the under-assessment plans. In addition, the criteria for unsatisfied dosimetric endpoints in the 5 poor-quality plans could typically be satisfied when the TPS-QC tool generated re-optimized plans without sacrificing other dosimetric endpoints. In addition to its feasibility and accuracy, the proposed TPS-QC tool is also user-friendly and easy to operate, both of which are necessary characteristics for clinical use.

## Introduction

Intensity-modulated radiation therapy (IMRT) has been widely accepted as a favorable treatment modality for multiple types of tumors [1–5]. Compared with conventional three-dimensional conformal radiotherapy (3D-CRT), IMRT can provide a higher conformal and more uniform dose distribution to the target while minimizing the dose to the surrounding normal tissues and organs at risk (OAR) [6–10]. An optimal plan should be created for every individual patient to ensure the best radiotherapy treatment efficacy; however, creating an optimal IMRT plan is complicated. Researches show that a clinically approved plan’s quality can vary significantly, depending on how experienced the planner is, how much time the planner spends on the plan, how difficult a particular planning job is, how good the communication between the physician and the planner is, the institution-specific planning protocols, and a number of other factors. [11–16]. High quality plans are hardly guaranteed for every single patient, while no plan quality evaluation tool is available under current clinical circumstances. Therefore, a tool that can easily and accurately conduct IMRT plan QC must be developed and afterwards widely applied as an essential component before the final plan is submitted.

The IMRT plan creation is mainly the process of searching optimal solutions with a dose optimization algorithm within given dosimetric goals [17–20]. Therefore, plan quality relies heavily on how optimal the given dosimetric goals are and how well the optimization algorithm explores the globally optimal solution. Wu et al (2009) introduced a method for quality control by flagging plans in which the patient geometry is similar but the structure doses are worse when matched against previous treatment plans. Subsequently, Moore et al (2011) built a mathematical model to predict achievable OAR dose-volume histograms (DVHs) using a sub-DVH concept. “Sub-DVH” describes the expected dose to an OAR’s sub-volume at a certain distance from the PTV surface and could be generated using a set of previous plans. By treating predictions as dosimetric goals, clinicians were able to improve on normal tissue sparing.

Although achievable dosimetric goals can be predicted ahead of time, a globally optimal solution is hardly guaranteed due to the limitations of currently available optimization algorithms. The solution domain is normally limited to a partial Pareto surface for current optimization algorithms in commercial treatment planning systems (TPS), which are organ-weighting factor-based systems. When the optimization objective functions are modified to voxel-weighting factor-based systems, the solution domain can be expanded to the entire Pareto surface, therefore increasing the probability of finding a more optimal plan [21, 22]. Therefore, a voxel-weighting factor-based plan re-optimization algorithm can be applied to perform IMRT plan quality control to ensure the best possible radiation treatment for each cancer patient.

Cotrutz and Xing [21, 22] first refined the traditional organ-weighting factor-based optimization objective function to be voxel based to increase the adjustment freedom of different volumes within each organ. These authors updated the optimizations iteratively and found that better trade-offs could be achieved among planning dosimetric endpoints than among traditional models. Later, Wu et al [23] proposed their own voxel-weighting factor-based models and mathematically proved that voxel-weighting factor-based optimization methods can carefully balance the trade-offs. Moreover, these authors found that re-optimizing original plans with voxel-based objective functions is equivalent to tuning the prescription dose in certain conditions. Later, Albin Fredriksson [24] established another voxel-weighting factor-based optimization model with constraints to enforce each voxel’s dose or the dose-volume histogram (DVH) of each organ to be at least as good as in the reference plan. By doing this, the original plan quality could automatically be improved.

Although the aforementioned methods have demonstrated the potential feasibility of applying voxel-weighting factor-based re-optimization algorithms to plan quality improvements, additional issues may need to be addressed in developing practical plan QC tools. First, the process of optimizing the algorithm search for the optimal solution is automated when certain voxel-weighting factors are given, but the voxel-weighting factors are manually determined. This makes it difficult to satisfy the high automation requirement for clinical application. Second, the emphasis on the above-mentioned re-optimization methods expands the optimization solution domain; however, the feasibility of applying these methods as a plan QC tool, such as the efficiency and accuracy of the evaluation, are not mentioned. Based on these observations, we incorporated additional modules and enhanced our own in-house re-optimization model to be a highly automated and accurate IMRT plan QC tool (TPS-QC tool). After importing an under-assessment plan, the TPS-QC tool was able to generate a potential optimal plan for the imported plan; then, by comparing the original plan with the optimized plan, the original plan quality could be determined. The plan quality determination is given in the form of a QC report, which can be automatically created within 2 minutes of when a plan is imported. In addition to the quality determination, the QC report also contains information supporting the results. Finally, the IMRT plan quality can be controlled by approving quality-passed plans and replacing quality-failed plans with TPS-QC-generated optimal plans.

## Methods and Materials

The proposed plan QC tool was designed within SCORE (Super Computer On-line Re-planning Environment), which is an in-house IMRT re-planning system capable of plan loading, CT to CT/CBCT rigid and deformable registration [25–27], plan re-optimization [28–32], and dose calculation [33]. The entire system was fully implemented on a graphic processing unit (GPU) to accelerate its performance and was developed to perform adaptive radiotherapy (ART) re-planning [34] and automated treatment planning. To enhance the SCORE system and make it a highly automated, effective and accurate IMRT plan QC tool, additional related modules were implemented. The QC workflow and feasibility, as well as the determination accuracy, were then tested and evaluated with both high- and poor-quality IMRT cases.

### 1. QC-related modules and integrated workflow

Additional modules, including IMRT planning protocol importation and plan quality report generation, were appended. The IMRT planning protocol importation module aimed to build an IMRT plan protocol database with multiple types of tumor sites and specific institution endpoint emphasis with corresponding criteria. From the user imported/chosen plan protocol, IMRT plan dosimetric endpoints and criteria can be obtained for further plan quality evaluations. Automated plan quality report generation comprised the main process and included the following steps: 1) launch plan protocol importation and SCORE system, including plan reading, plan re-optimization, leaf sequencing and final dose calculation based on the user-imported under-assessment plan to generate a deliverable SCORE re-optimized plan; 2) extract dosimetric endpoint values from both the under-assessment plan and the SCORE re-optimized plan as the plan quality indicators; 3) compare every endpoint value with the aforementioned criterion, and fill “green”/“red” when a value satisfies or does not satisfy the corresponding criterion; 4) compare every endpoint value from the under-assessment plan with that from the SCORE re-optimized plan, and mark “red” when the value poorly satisfies the criterion (here, less satisfaction of the value indicates lower PTV coverage or the OAR receiving a higher dose); 5) determine the under-assessment plan quality as “passed”/“failed” when performing a Wilcoxon matched pairs signed ranks test between data arrays constructed by all endpoint values from the under-assessment plan and the SCORE re-optimized plan (a determination of “failed” is assigned when a significant difference is observed; otherwise, a determination of “passed” for the quality determination is assigned to the under-assessment plan); and 6) generate a complete plan quality report, including the under-assessment plan vs. the SCORE re-optimized plan visual DVH comparison, dosimetric endpoint value table, and final plan quality determination.

**Fig 1:**
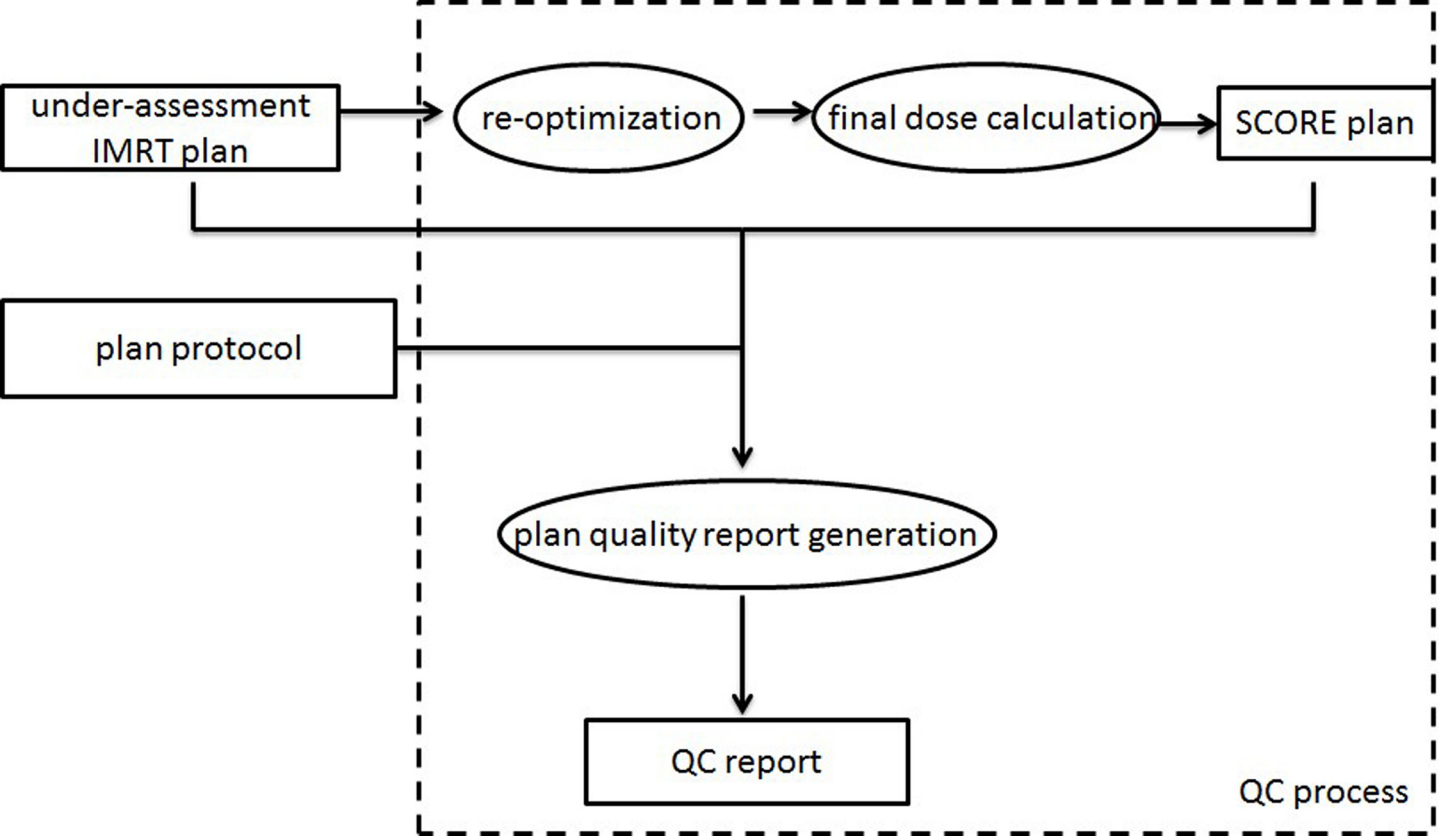
Workflow for the IMRT TPS-QC tool.

The integrated plan QC workflow is shown in Fig 1 (Fig 1). Plans needing to be evaluated are referred to as under-assessment plans, and plans after SCORE re-optimization are called SCORE re-optimized plans. To compare the plan quality fairly, we first re-calculated the original clinical exported plan using the dose engine within the SCORE to obtain the under-assessment plans. Before launching the re-optimization algorithm, a plan protocol should be imported or selected from the database to emphasize plan dosimetric endpoints and criteria. After an under-assessment plan is imported into SCORE, a SCORE re-optimized plan will be generated automatically. The developed automatic plan quality report generation module will then be used to give the final plan quality determination and declare whether the plan quality is satisfactory or unsatisfactory. In addition, a SCORE re-optimized plan is offered by the TPS-QC tool to replace the under-assessment plan when necessary. To better support the plan quality determination, a detailed plan quality comparison is demonstrated with both DVH and dosimetric endpoint values between the under-assessment plan and the SCORE re-optimized plan.

### 2. Plan re-optimization

Plan re-optimization is one of the key steps in our proposed TPS-QC tool. In the SCORE system, we have developed an in-house voxel-weighting factor-based IMRT plan re-optimization model and mathematically proven its ability to explore a much larger Pareto surface than does the traditional organ-weighting factor-based model [32, 34]. The detailed optimization objective function is formulated below:

$$F_{s}\left(d\right)=\sum_{s\in T}\alpha_{s}^{-}\sum_{j\in v_{s}}\left(w_{j}^{-}\cdot {\left(max\left\{0，\;P_{j}-d_{j}\right\}\right)}^{2}\right)+\sum_{s\in C}\alpha_{s}^{+}\sum_{j\in v_{s}}\left(w_{j}^{+}\cdot {\left(max\left\{0，\;d_{j}-P_{j}\right\}\right)}^{2}\right)$$

where $\alpha_{s}^{-}$ and $\alpha_{s}^{+}$ are under-dose and over-dose organ-weighting factors, while $w_{j}^{-}$ and $w_{j}^{+}$ are those factors for voxels. *P*_*j*_ denotes the prescription dose for the target or threshold dose for critical structures. Given a set of organ- and voxel-weighting factors, the optimal solution could be obtained using a gradient projection method [28]. Two iteration loops were facilitated in the optimization algorithm: 1) an inner loop, namely the coarse-adjusting stage, in which the organ-weighting factors are adjusted based on the DVH difference between the calculated plan and the reference plan; and 2) the outer loop, namely the fine-tuning stage, in which the voxel-weighting factors are adjusted individually within one organ to obtain a more desirable DVH.

With the help of these two iteration loops, voxel-weighting factors can be obtained automatically under a reference DVH. This reference DVH constrains the newly generated plan, which should be at least as good as the reference while reduce the OAR dose as much as possible. For the purposes of plan QC, the reference plan chosen was the under-assessment plan itself. If the reference plan is feasible but not Pareto-optimal, the algorithm can generate a Pareto-optimal plan with DVHs better than those in the reference plan. The feasibility of this method has been validated and applied to ART and automated treatment planning for different tumor sites [34]. Efficiency was achieved with a total optimization time of less than 30 sec.

Issues such as multi-leaf collimator (MLC) leaf sequencing and MLC modeling were fully considered and implemented, and therefore, a SCORE-generated re-optimized plan is a clinically deliverable IMRT plan. For dose calculation, an in-house GPU-based finite size pencil beam (FSPB) algorithm with 3D density correction was carried out, and its accuracy was validated by comparing it with a Monte Carlo method and analytical anisotropic algorithm (AAA) using a commercial Eclipse system (Varian Medical Systems, Palo Alto, California, US) [33].

### 3. TPS-QC tool evaluation

QC-related module functions and feasibility were evaluated, as were the plan quality determination efficacy and accuracy. Twenty-five clinically approved cervical cancer patient IMRT plans from the UC San Diego Moores Cancer Center were randomly selected. The prescription dose was 4,500 cGy for patients with intact cervix cancer and 5,040 cGy for post-operative patients in 180 cGy daily fractions over 5–5.5 weeks. PTV and critical structures were considered to demonstrate plan quality representative organs. These critical structures included the bowel, bladder, rectum, bone marrow and femoral head. All treatment plans were created with the Eclipse system by experienced dosimetrists with sufficient time and effort. The International Evaluation of Radiotherapy Technology Effectiveness in Cervical Cancer (INTERNECC) protocol was employed in this study as an example. The protocol-announced dosimetric endpoints and criteria are listed in detail in Table 1 (Table in S1 Protocol).

**Table 1:** Dosimetric endpoints and criteria for assessing IMRT plan quality.

organs		endpoints	criteria
		V_99_	> = 90%
		V_90_	> = 99%
PTV		V_97_	> = 97%
		V_115_	<1%
		V_110_	<10%
		D_max_overall_	In PTV
OAR			
	Bowel	V_45Gy_	<250cc
		D_max_	<115%
	Rectum	D_max_	<115%
	Bone Marrow	V_10Gy_	<90%
		V_20Gy_	<75%
	Bladder	D_max_	<115%
	Femoral Head	D_max_	<115%

*Abbreviations*:

Vx = percentage volume receiving x% of prescription dose

D_max_overall_ = maximum dose in plan

V_x Gy_ = percentage volume receiving x Gy

D_max_ = maximum dose in each organ; cc = cm^3^

To further verify the effectiveness and robustness of the proposed TPS-QC tool, another 5 poor-quality IMRT plans were generated to undergo the QC workflow. These generated plans were created with very loose dose volume objects (i.e., 40%, 90% and 75% for V_45Gy_ of bowel, V_10Gy_ of bone marrow and V_20Gy_ of bone marrow, respectively) when performing the plan optimization procedure in the Eclipse system. By doing this, we sought to verify that when the under-assessment plans show very poor quality, the TPS-QC tool can accurately identify and automatically improve them.

Therefore, there were 30 cervical patient IMRT plans in total with which to test the proposed TPS-QC tool. After performing the plan QC function, we used a Wilcoxon matched pairs signed ranks test to analyze the significant difference between the under-assessment plan and the new SCORE plan quality. Plan quality was reflected by the extracted dosimetric endpoint value array.

## Results

### 1. Plan QC report

For each under-assessment IMRT plan, a specific QC report was generated that compared the results to the new SCORE re-optimized plan. This report contains two parts: the comparison details and the plan quality determination. The quality comparison details between the under-assessment IMRT plan and the SCORE re-optimized plan are presented in terms of DVH and plan dosimetric endpoint values for every PTV and OAR. Plan dosimetric endpoint values are filled in green when the protocol-announced criterion is satisfied; otherwise, these values are filled in red. In addition, the under-assessment plan dosimetric endpoint values are marked in red when further improvement could be made after comparison with the SCORE re-optimized plan; otherwise, they are marked in black.

The entire QC process was completed within 2 minutes for each case. Protocol importation, QC report generation and other QC-related modules worked well and were completed within seconds. The QC report can be viewed as a PDF file and saved when necessary. The SCORE re-optimized plan is generated automatically and can be exported to replace the under-assessment plan when poor quality is identified.

### 2. Evaluation of clinically approved plans

**Fig 2:**
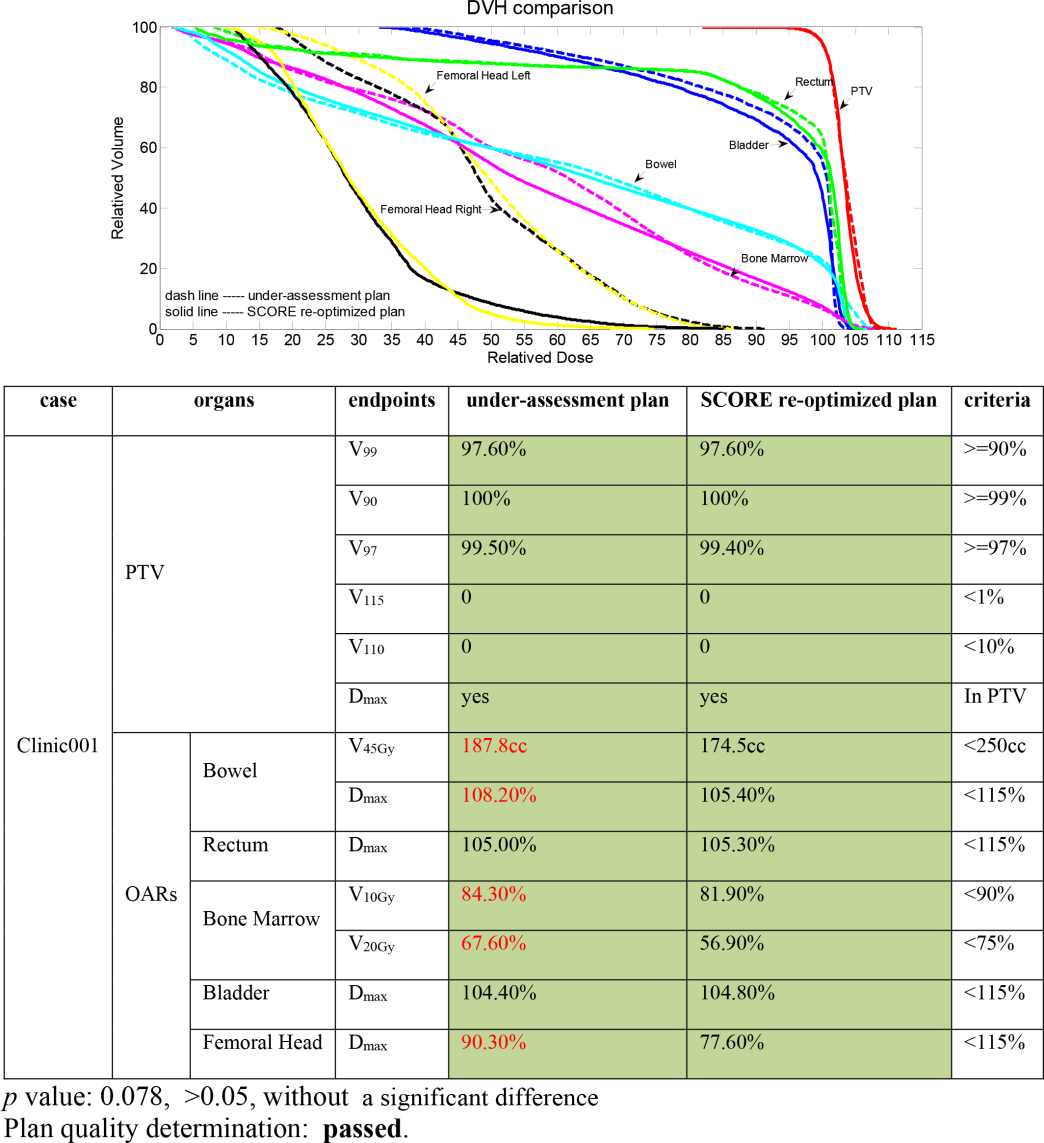
A QC report example for one clinically approved cervical cancer IMRT plan.

Fig 2 illustrates a QC report example from one clinical cervical cancer IMRT case (Fig 2). In this report, green was filled for all dosimetric endpoints from both the under-assessment plan and the SCORE re-optimized plan, indicating that both plans effectively satisfied the protocol criteria. Moreover, red was marked for several dosimetric endpoint values of the under-assessment plan, including V_45Gy_ and D_max_ of the bowel, V_20Gy_ of the bone marrow and D_max_ of the femoral head, indicating that further dose sparing could be obtained for these particular dosimetric endpoints after re-optimization. The overall *p* value between the under-assessment plan and the SCORE re-optimized plan dosimetric endpoint value array was greater than 0.05, showing no significant difference and indicating that the under-assessment plan quality passed, although several dosimetric endpoints could be further improved.

The same processes were conducted for the other 24 clinically approved cervical cancer IMRT plans, and a QC report was generated for each one. The results showed that the overall *p* value for each under-assessment plan and SCORE re-optimized plan dosimetric endpoint value array pair was mostly greater than 0.05, showing no significant difference and indicated that the plan quality passed; unless one of them with a *p* value of 0.047(case “Clinic010”), less than 0.05, demonstrating significant difference (detailed data could be seen in the S1 Dataset, sheet “WilcoxonTestForClinicalPlans”).

**Fig 3:**
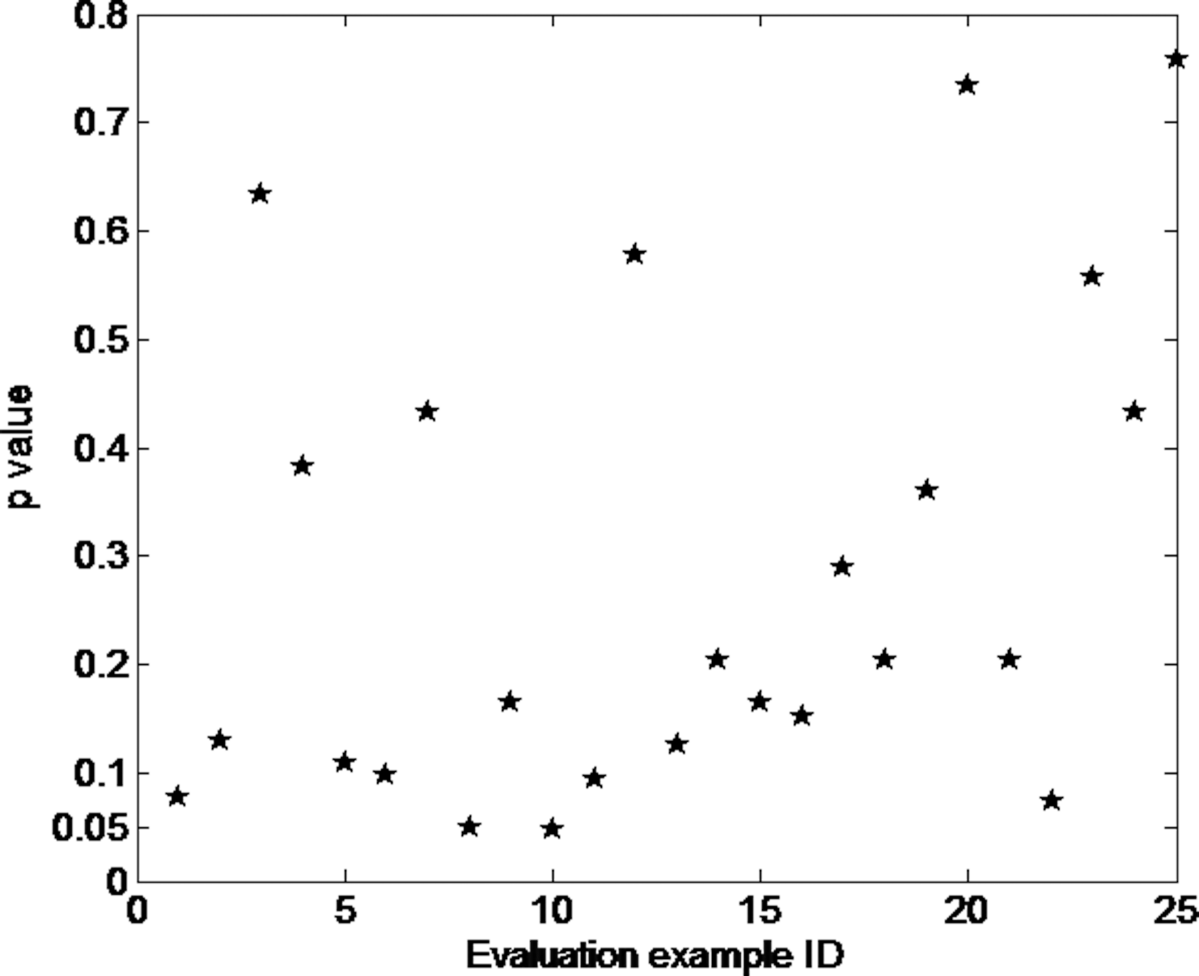
Scatter plot of *p* values obtained from clinical cervical cancer IMRT plans.

For this particular clinically approved but with “failed” plan quality case, all dosimetric endpoints from both the under-assessment plan and the SCORE re-optimized plan satisfied the protocol criteria. Meanwhile, most dosimetric endpoint values of the under-assessment plan were improved after re-optimized, including V_99_ of the PTV, from 95.6% to 96.8% for original plan and after re-optimization respectively; D_max_ of the bowel, from 108.4% to 103.2%; D_max_ of the rectum, from 105.6% to 103.7%; V_10Gy_ and V_20Gy_ of the bone marrow, from 84.4% and 68.2% to 83.6% and 66.1% respectively, D_max_ of the bladder, from 108.9% to 104.5% and D_max_ of the femoral head, from 98.5% to 66.5%. The scatter plot of *p* values obtained for the 25 clinical approved cervical cancer IMRT plans can be seen in Fig 3 (Fig 3). The mean *p* value was 0.282, and the standard variance was 0.222.

Although the plan quality passed for most of these 25 clinically approved cervical cancer IMRT plans, a larger difference was more frequently obtained for the OAR dosimetric endpoints than for the PTVs, implying that better dose sparing may be achieved in OAR than in PTVs. Moreover, the DVH curves of the SCORE re-optimized plans satisfied the dosimetric criteria more frequently than did the under-assessment plans, which indicates the better quality of the SCORE re-optimized plans and also the opportunity for improvement of the under-assessment IMRT plans.

### 3. Evaluation of poor-quality plans

**Fig 4:**
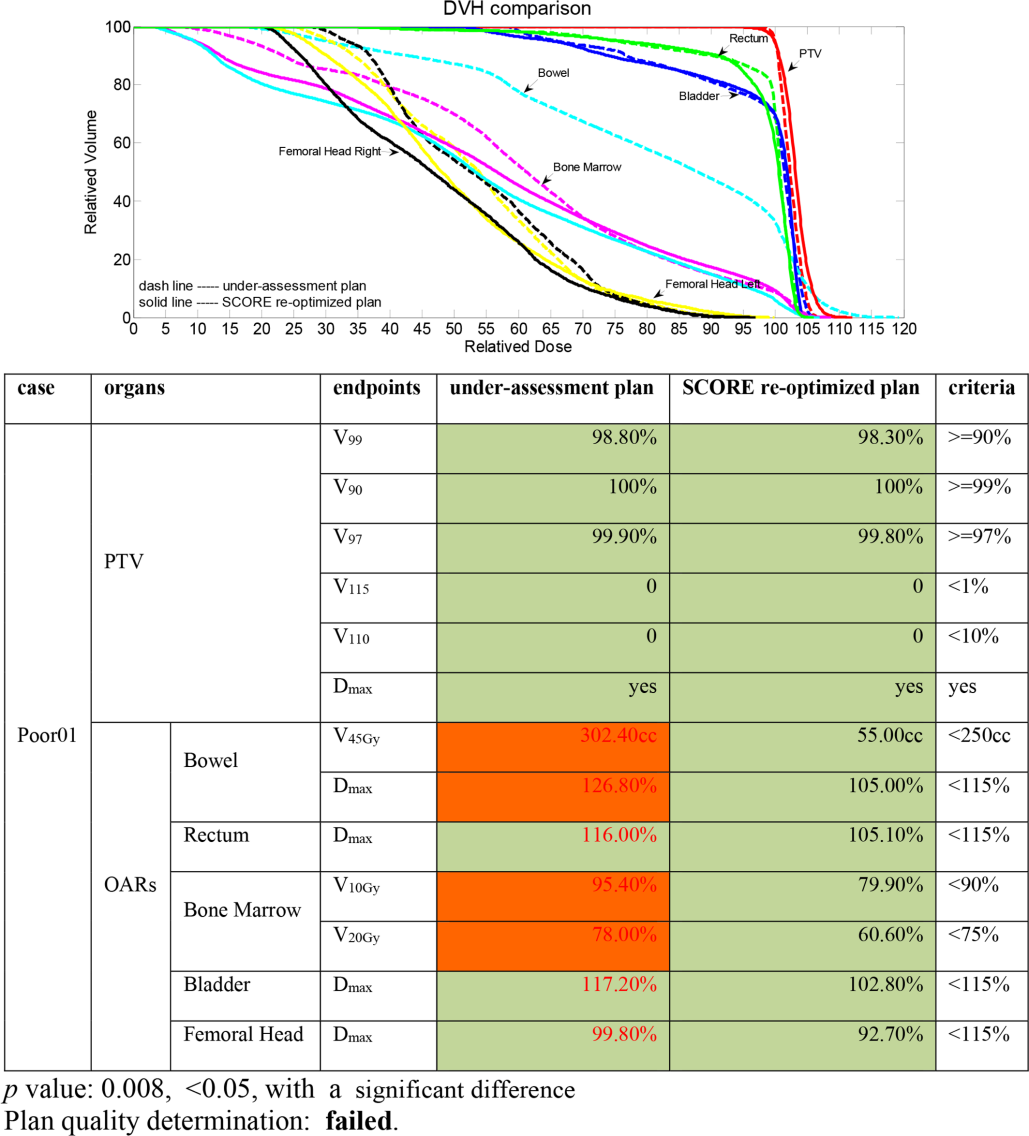
A QC report example for a poor-quality plan.

The TPS-QC tool also processed the 5 manually generated poor-quality cervical cancer patient IMRT plans. Fig 4 illustrates a QC report derived from one of those plans (Fig 4). In this report, four dosimetric endpoint criteria were not satisfied for the under-assessment plan and are filled in red, namely, V_45Gy_ and D_max_ of the bowel and V_10Gy_ and V_20Gy_ of the bone marrow. For the corresponding SCORE re-optimized plan, these unsatisfied dosimetric endpoints became satisfactory without sacrificing the performance of any other dosimetric endpoints. More specifically, the dosimetric endpoints were reduced by 247.4 cc and 21.8% of the prescription dose for bowel V_45Gy_ and D_max_ and 15.5% and 17.4% of the structure volume for bone marrow V_10Gy_ and V_20Gy_, respectively. Differences in other OAR dosimetric endpoints were observed to decrease by 10.90%, 14.40% and 7.10% for D_max_ of the rectum, D_max_ of the bladder and D_max_ of the femoral head, respectively. The PTV coverage differed slightly between the under-assessment plan and the SCORE re-optimized plan, with reductions of 0.50% and 0.10% for V_99_ and V_97_, respectively, and the same was true for other dosimetric endpoints (data are shown in the S1 Dataset, sheet “WilcoxonTestForPoorQualityPlans”). The overall *p* value for the under-assessment plan and SCORE re-optimized plan dosimetric endpoint value array pair was less than 0.05, showing a significant difference and indicating that the plan quality failed. In this case, users could choose to replace the under-assessment plan with the SCORE re-optimized plan or to adjust the under-assessment plan manually until the updated plan quality was found to pass.

In the 4 QC reports derived from the 4 other poor-quality IMRT plans, the criteria that were unsatisfactory in the dosimetric endpoints in the under-assessment plans became satisfactory in the corresponding SCORE re-optimized plans without sacrificing other dosimetric endpoints, indicating both the possibility of improving the under-assessment plans and the high accuracy of the TPS-QC tool. In total, the overall *p* value for each under-assessment plan and SCORE re-optimized plan dosimetric endpoint value array pair was less than 0.05 (on average 0.016 ± 0.006) (detailed data could be seen in the S1 Dataset, sheet “WilcoxonTestForPoorQualityPlans”), showing a significant difference and indicating that the plan quality failed.

## Discussion

Current studies for plan quality control/assessment are mainly focused on previous knowledge learning to correlate patient geometry and its potential plan dosimetry. The predicted plan dosimetry (i.e., DVH) is then obtained by applying the correlation model to a new patient geometry as a standard of the quality acceptable plan. Since correlations are more easily explored when learnt from similar plans, the experience-based methods conduce a model exclusiveness for each tumor site or even institution. Unlike the experience-based model is usually tumor site or even institution specific, the proposed re-optimization based TPS-QC tool gives a more general solution that most of IMRT plans are applicative. In addition to this, the re-optimization stratagem can also avoid the process of building up treatment plan training databases, which would be a rigorous issue when cases are insufficient at some initial stages.

No matter the experience-based method or the re-optimization stratagem, both of them can help to establish a new standard of potential higher quality treatment plan, though in different ways of either predicting optimal dosimetric goals or expanding the solution domain. As previously mentioned, the IMRT plan creation is mainly the process of searching optimal solutions with a dose optimization algorithm within given dosimetric goals, consequently an idea high quality standard is more favorably released by considering not only patient specific optimal target but also a possibly complete solution domain. Therefore, the experience-based method and the re-optimization stratagem are not antipathetic but mutual that they should be combined to collectively ensure cancer patient’s specific high quality treatment.

From these 25 assumed high quality clinically approved IMRT plans, one of them was tested with significant difference and hence identified as with “failed” quality. After having this particular plan re-checked with our experienced dosimetrist, we found that no criterion is created for neither the left femoral head nor the right femoral head. This caused that the D_max_ of femoral head obtained a remarkable improvement after further optimized by our TPS-QC tool, namely from 98.5% to 66.5%, for original plan and the SCORE re-optimized plan respectively. Besides, since a clinically approved IMRT plan is created from a complicated planning process, as previously mentioned, it is possible that few plans are generated with undesirable quality.

Though our developed plan quality control tool shew high consistency with the actual IMRT plan quality, and can complete the whole evaluation process automatically, there remain some limitations for further investigation and improvement. For instance, we incorporated a two-stage automated voxel-weighting factor tuning strategy in the plan optimization, of which the weighting factor tuning is a function of previously and currently DVH area difference. Admitting that the weighting factor is fully automatically adjusted, the rule of adjustment is intuitive. Underground scientific correlations behind is still not clear so far [34]. As a consequence, not only the tuning method but also the iteration limit, which is set to restrict the iterations for solving an optimization objective function, would be affected, thus impact the solving accuracy and its efficiency.

Our future work aims to apply the TPS-QC tool to other tumor sites for clinical use. Future efforts will focus on tumor site-specific optimization and application improvements, including enabling interactions with commercial TPSs and incorporating feedback from planners and physicians. In addition, another challenging project is to take the potential QA failures into account so that the optimized higher quality plan could be ensured. Although the leaf sequencing has already been considered and the dose engine (FSPB) has been customized to the commercial TPS at the current stage, more improvements should be made, such as considering more machine limitations during the leaf transmission and incorporating the Monte Carlo dose calculation algorithm into the SCORE system.

## Conclusions

In this work, we practically apply a voxel-weighting factor-based plan re-optimization method to perform treatment plan quality control. Based on this approach, we integrated a highly automated and potentially clinically useful IMRT plan QC tool. The feasibility and accuracy of the proposed TPS-QC tool were verified using 25 clinically approved cervical cancer patient IMRT plans and 5 poor-quality IMRT plans. The integrated TPS-QC tool is user-friendly and easy to operate. In addition, the under-assessment plan QC report can be created automatically within 2 minutes of being imported. Furthermore, the TPS-QC tool can generate a corresponding re-optimized plan that can replace the failed under-assessment plan. This replacement can not only help improve treatment plan quality but also reduce the manual effort needed to adjust the plan, thereby laying the foundation for the application of the TPS-QC tool in clinics.

## Supporting Information

S1 Dataset Plan dosimetric endpoint values (original vs. SCORE re-optimized). (XLSX)

S1 Protocol The INTERTECC protocol. (PDF)
