# Supplementary material for: An Automated Treatment Plan Quality Control Tool for Intensity-Modulated Radiation Therapy Using a Voxel-Weighting Factor-Based Re-Optimization Algorithm
Source: PLoS One. 2016 Mar 1;11(3):e0149273. doi: 10.1371/journal.pone.0149273 (PMC4773182; doi:10.1371/journal.pone.0149273)
Supplement: S1 Protocol — (PDF) [file pone.0149273.s002.pdf]

INTERNATIONAL EVALUATION OF RADIOTHERAPY TECHNOLOGY EFFECTIVENESS IN CERVICAL CANCER (INTERTECC)

PHASE II/III CLINICAL TRIAL OF INTENSITY MODULATED RADIATION THERAPY WITH CONCURRENT CISPLATIN FOR STAGE I-IVA CERVICAL CARCINOMA

Activation Date: May 1, 2011
Version Date: November 1, 2012

IRTOC Headquarters
Attention: INTERTECC Study Coordinator (irtoc@ucsd.edu)
Center for Advanced Radiotherapy Technologies
3855 Health Sciences Drive #0843
La Jolla, CA 92093-0843
(858)246-0544 / Fax: (858)822-5568

This protocol was designed and developed by the International Radiotherapy Technologies and Oncology Consortium (IRTOC) and is intended to be used only in conjunction with IRB approval for study entry. No other use or reproduction of this protocol is authorized or endorsed by IRTOC.

INVESTIGATORS (UCSD)

Radiation Oncology (UC San Diego)

Loren K. Mell, M.D. (Principal Investigator)
University of California San Diego
Center for Advanced Radiotherapy Technologies
3855 Health Sciences Drive #0843
La Jolla, CA 92093-0843
(858) 246-0471 / Fax: (858)822-5568
lmell@ucsd.edu

Catheryn M. Yashar, M.D.
University of California San Diego
Center for Advanced Radiotherapy Technologies
3855 Health Sciences Drive #0843
La Jolla, CA 92093-0843
(858)822-3507
cyashar@ucsd.edu

Arno J. Mundt, M.D.
University of California San Diego
Center for Advanced Radiotherapy Technologies
3855 Health Sciences Drive #0843
La Jolla, CA 92093-0843
(858) 246-6520
amundt@ucsd.edu

Medical Physics (UC San Diego)

Steve Jiang, Ph.D. (IGRT)
University of California San Diego
Center for Advanced Radiotherapy Technologies
3855 Health Sciences Drive #0843
La Jolla, CA 92093-0843
(858)822-5129
sbjiang@ucsd.edu

Todd Pawlicki, Ph.D. (QA)
University of California San Diego
Center for Advanced Radiotherapy Technologies
3855 Health Sciences Drive #0843
La Jolla, CA 92093-0843
(858) 822-6058
tpawlicki@ucsd.edu

Dan Scanderbeg, Ph.D. (Brachytherapy)
University of California San Diego
Center for Advanced Radiotherapy Technologies
3855 Health Sciences Drive #0843
La Jolla, CA 92093-0843
(858) 822-7928
dscanderbeg@ucsd.edu

William Song, Ph.D. (IGRT)
University of California San Diego
Center for Advanced Radiotherapy Technologies
3855 Health Sciences Drive #0843
La Jolla, CA 92093-0843
(858) 822-7928
wysong@ucsd.edu

Gynecologic Oncology (UC San Diego)

Steve Plaxe, M.D.
University of California San Diego
Moores Cancer Center
3855 Health Sciences Drive #0987
La Jolla, CA 92093
(858) 822-6199
splaxe@ucsd.edu

Michael McHale, M.D.
University of California San Diego
Moores Cancer Center
3855 Health Sciences Drive #0987
La Jolla, CA 92093
(858) 822-6275
mtmchale@ucsd.edu

Cheryl Saenz, M.D.
University of California San Diego
Moores Cancer Center
3855 Health Sciences Drive #0987
La Jolla, CA 92093
(858) 822-6199
csaenz@ucsd.edu

Biostatistics

Ronghui (Lily) Xu, Ph.D.
University of California San Diego
Department of Family and Preventive Medicine
9500 Gilman Drive
La Jolla, CA 92093-0112
(858) 534-6380
rxu@ucsd.edu

Mike Donohue, Ph.D.
University of California San Diego
Department of Family and Preventive Medicine
9500 Gilman Drive
La Jolla, CA 92093-0112
(858) 534-6380
mdonohue@math.ucsd.edu

Bioinformatics

Aziz Boxwala, M.D., Ph.D.
Head, Section on Clinical Informatics
University of California San Diego
Department of Medicine
Division of Biomedical Informatics
9500 Gilman Dr. #0728
La Jolla, CA 92093-0728
(858) 822-3348
Fax: (858) 822-7685
aboxwala@ucsd.edu

Enrolling Centers (Alphabetical Order):

University Hospital Hradec Králové

Igor Sirák, M.D., Ph.D.
Department of Oncology and Radiotherapy
University Hospital Hradec Králové
Hradec Králové, Czech Republic
pigic@seznam.cz

Linda Kasaova, M.Sc.
Department of Oncology and Radiotherapy
University Hospital Hradec Kralove
Sokolska 581, 500 05
Hradec Kralove, Czech Republic
kasaova@fnhk.cz
phone: 00420 495 833 373

Xijing Hospital

Mei Shi, M.D.
Director, Department of Radiation Oncology,
Xijing Hospital, Fourth Military Medical University,
15, Changle West Road, Xi'an, Shannxi province
Code: 710032
Tel: 86-29-84775425 (office), 86-29-84775432 (office), 13609249669 (cell phone)
mshi82@fmmu.edu.cn; meishi82@188.com

Lichun Wei, M.D., Ph.D.
Department of Radiation Oncology
Xijing Hospital, The Fourth Military Medical University
17#, Changle West Road, Xi'an, Shaanxi, P.R.China, 710032
Tel: 86-29-84775432 (office) / 13572019003 (cell)
weilichun@fmmu.edu.cn

IMAGING TECHNOLOGY AND QA

Jeff Michalski, M.D.
Siteman Cancer Center
Center for Advanced Medicine
4921 Parkview Place, LL
St. Louis, MO 63110
314-747-7236
Fax: 314-747-9557
jmichalski@radonc.wustl.edu

Walter Bosch, D.Sc.
Research Assistant Professor in Radiation Oncology
Image-guided Therapy Center
4511 Forest Park Blvd., Suite 200
St. Louis, MO 63108
314-747-5414
wbosch@radonc.wustl.edu

William Straube, M.S.
Image-guided Therapy Center

David Followill, Ph.D.
Department of Radiation Physics

4511 Forest Park Blvd., Suite 200
St. Louis, MO 63108
314-747-5414
wstraube@radonc.wustl.edu

Division of Radiation Oncology
University of Texas MD Anderson Cancer Center
1515 Holcombe Blvd.
Houston, TX 77030
dfollowi@mdanderson.org

Jessica Lowenstein, M.S.
Department of Radiation Physics
Division of Radiation Oncology
University of Texas MD Anderson Cancer Center
1515 Holcombe Blvd.
Houston, TX 77030
jlowenst@mdanderson.org

DATA SAFETY AND MONITORING COMMITTEE

Mary Ann Rose, M.D. (Chair)
University of California San Diego
1200 Garden View Rd, Suite 210
Encinitas, CA 92024
Phone : 858/246-0500
mrose@ucsd.edu

Roger Rice, Ph.D.
University of California San Diego
3855 Health Sciences Drive #0843
La Jolla, CA 92093-0843
(858) 822-6040
rrice@ucsd.edu

Laura Cerviño, Ph.D.
University of California San Diego
Center for Advanced Radiotherapy Technologies
3855 Health Sciences Drive #0843
La Jolla, CA 92093-0843
(858) 246-6520
lcervino@ucsd.edu

Jong Hyeon-Jeong, Ph.D.
University of Pittsburgh
318B Parran Hall, 130 DeSoto Street
Pittsburgh, PA 15261
(412) 624-8549
jeong@nsabp.pitt.edu

DATA QUALITY AND AUDITING

Jong Hyeon-Jeong, Ph.D.
University of Pittsburgh
318B Parran Hall, 130 DeSoto Street
Pittsburgh, PA 15261
(412) 624-8549
jeong@nsabp.pitt.edu

QUALITY OF LIFE ANALYSIS

Clement K. Gwede, PhD, MPH, RN
Assistant Member & Assistant Director of Diversity,
Moffitt Cancer Center Division of Population
Sciences, Dept. of Health Outcomes & Behavior
12902 Magnolia Dr., MRC-CANCONT
Tampa, FL 33612
PH: 813-745-3052
FX: 813-745-6525
clement.gwede@moffitt.org

TABLE OF CONTENTS

1.0 Schema
2.0 Eligibility Checklist
3.0 Registration Worksheet
4.0 Introduction
5.0 Objectives
6.0 Population and Eligibility Criteria
7.0 Pretreatment Evaluations/Management
8.0 Registration Procedures
9.0 Radiation Therapy
10.0 Drug Therapy
11.0 Surgery
12.0 Other Therapy
13.0 Pathology and Tissue/Specimen Submission
14.0 Patient Assessments, Follow-Up, and Data Collection Procedures
15.0 Patient Safety and Ethical Considerations
16.0 Statistical Considerations
17.0 References
18.0 Appendices
18.1 Appendix I: Sample Consent Form
18.2 Appendix II: FIGO Staging System
18.3 Appendix III: Performance Status Scoring System
18.4 Appendix IV: Quality of Life Assessment (QOLA) Form
18.5 Appendix V: Physics Questionnaire Form (PQF)

1.0 SCHEMA

Design: **Phase II/III Clinical Trial**

Sample Size: 91 (Phase II); 334 (Phase III)

Population / Eligibility (Section 6.0):

Biopsy-proven Stage I-IVA Invasive Cervical Carcinoma

Post-hysterectomy with high-risk features

OR

Locally advanced, inoperable / intact cervix

Radiation Therapy (Section 9.0):

**Phase II:**	**IMRT, 45.0 (intact) or 50.4 Gy (postoperative high-risk) in 1.8 Gy daily fractions over 5-5.5 weeks**
**Phase III, Arm A:**	**IMRT as above**
**Phase III, Arm B:**	**Conventional RT, 45.0 (intact) or 50.4 Gy (postoperative high-risk) in 1.8 Gy daily fractions over 5-5.5 weeks**
**All Arms:**	**Intracavitary Brachytherapy (Optional for Postoperative Patients)**

Chemotherapy (Section 10.0):

**All Patients:**	**Cisplatin, 40 mg/m^2 weeks 1-5 of external beam RT**

Surgery (Section 11.0):

**All Patients:**	**Hysterectomy or Biopsy**

Strata for Pre-Planned Subgroup Analysis:

- **-Postoperative vs. Intact Cervix**
- **-HIV Positive Status**
- **-Use of pre-treatment image-guidance (PET or MRI)**
- **-Use of daily in-room IGRT (planar or volumetric)**
- **-For Intact Cervix Patients:**
  - **-Point-Directed vs. Volume-Directed Brachytherapy**
  - **-LDR vs. HDR Brachytherapy**

Sub-Studies:

(1) Modeling Hematologic Toxicity using High Dimensional Data Analysis

(2) Daily kV Cone Beam Computed Tomography (CBCT) to Determine Optimal Planning Margins

(3) Effectiveness of Functional Bone Marrow Sparing IMRT

2.0 ELIGIBILITY CHECKLIST

(Y) 1. Does the patient have biopsy-proven, invasive primary squamous cell carcinoma, adenocarcinoma, or adenosquamous carcinoma of the cervix?
(Y) 2. Does the patient have a biopsy result positive for carcinoma within 60 days prior to registration?
(Y) 3. Does the patient have FIGO clinical stage I-IVA cervical cancer (Appendix II)?
(N) 4. Does the patient have clinical, radiographic, or pathologic evidence of para-aortic or inguinal nodal and/or distant metastasis?
(N) 5. Does the patient have radiographic or pathologic evidence of gross (unresected) pelvic nodal metastasis (see section 6.2.4)?
(Y) 6. Has the patient had a pelvic examination within 14 days prior to registration?
(Y) 7. Has the patient had an X-ray, CT scan, or PET/CT of the chest within 42 days prior to registration?
(Y) 8. Has the patient had a CT scan, MRI, or PET/CT of the pelvis within 42 days prior to registration?
(Y) 9. Is the patient's Karnofsky Performance Status ≥ 60 (Appendix III)?
(Y) 11. Is the patient ≥ 18 years of age?
(Y) 12. Has the patient met all the lab requirements as described in Section 6.1.10?
(Y) 13. If the patient is of child bearing potential, has she had a negative serum pregnancy test?
(Y) 14. If the patient is of child bearing potential, did she agree to practice effective birth control throughout the treatment phase of the study?
(N) 15. Is the patient pregnant or lactating?
(Y/NA)16. If clinical suspicion of AIDS, must have CD4+ T cell count > 200 per µL of blood and >14% of all lymphocytes
(Y/N) 17. Does the patient have a history of a prior invasive malignancy (with the exception of non-melanomatous skin cancer)?
(Y) If yes, has the patient been disease free for greater than three years?
(N) 18. Has the patient had prior systemic chemotherapy for cervical cancer?
(N) 19. Has the patient had prior radiation therapy to the pelvis or abdomen that would result in overlap of radiation therapy fields?
(N) 20. Has the patient had unstable angina and/or congestive heart failure requiring hospitalization within the last 6 months?
(N) 21. Has the patient had a myocardial infarction within the last 6 months?
(N) 22. Does the patient have an acute infection requiring antibiotics at the time of registration?
(N) 23. Does the patient have a Chronic Obstructive Pulmonary Disease or other respiratory illness requiring hospitalization or precluding study therapy at the time of registration?
(N) 24. Does the patient have hepatic insufficiency resulting in clinical jaundice and/or coagulation defects?
(N) 25. Does the patient have uncontrolled diabetes?
(N) 26. Does the patient have uncompensated heart disease or uncontrolled blood pressure per Section 6.2.6.7?
(N) 27. Does the patient have a $CD4^+$ T cell count < 200 per µL of blood or <14% of all lymphocytes?
(N) 28. Does the patient have any other immunocompromised status (e.g., organ transplant or chronic glucocorticoid use)?
(Y) 29. Did the patient sign a study specific informed consent prior to study entry?

IRTOC Institution #______________

Case # ________________________

3.0 REGISTRATION WORKSHEET

1. Name of person registering the case
(Y) 2. Has the Eligibility Checklist been completed and the patient determined to be eligible?
(Y) 3. Has the patient signed informed consent?
4. Patient's Initials (First Middle Last)
5. Verifying Physician
6. Patient's ID Number
7. Date of Birth
8. Race (see Appendix IV, section 18.4)
9. Ethnic Category (Hispanic or Latino; Not Hispanic or Latino; Unknown) (see Appendix IV)
10. Gender
11. Patient's Country of Residence
12. Zip Code (U.S. Residents)
13. Patient's Insurance Status
14. Will any component of the patient's care be given at a military or VA facility?
15. Calendar Base Date
16. Registration date
(Y/N) 17. Does the patient consent to be contacted for future research?
18. Gynecologic Oncologist's Name

The Eligibility Checklist must be completed in its entirety prior to registration. The completed, signed, and dated checklist used at study entry must be retained in the patient's study file and will be evaluated during an audit.

Completed by____________________________Date _______________________

***THIS FORM SHOULD BE TURNED IN AT THE TIME OF REGISTRATION**

4.0 INTRODUCTION

4.1 Background of Cervical Cancer

Cervical cancer is a leading cause of cancer mortality in women [1-2]. Despite the successes of screening and vaccination, a large proportion of women fail to comply with preventative measures and present with locally advanced stages of disease. Concurrent chemotherapy and radiation therapy (RT) is the standard treatment approach for patients with locally advanced cervical carcinoma. With standard cisplatin-based chemoradiotherapy, acute and late toxicity remain significant problems, and the incidence of locoregional failure, distant metastasis, and cancer mortality remain high [3-10]. Therefore, strategies to reduce toxicity and permit treatment intensification are needed.

4.2 Efficacy and Toxicity of Chemoradiation for Cervical Cancer

Multiple randomized controlled trials have established concurrent cisplatin-based chemoradiotherapy as the standard of care for locally advanced cervical cancer [3-8]. The addition of concurrent cisplatin to radiotherapy (RT) increases pelvic control, disease-free survival (DFS) and overall survival; however, 5-year DFS and overall survival are still only approximately 60% and 5-year pelvic failure is approximately 30% [3-10]. Moreover, acute gastrointestinal (GI) and hematologic toxicity are increased. Approximately 30% of patients will experience acute grade ≥ 3 toxicity, predominantly GI and hematologic [3-10].

Many studies have investigated multiagent chemotherapy as a means of intensifying treatment. For example, promising results have been seen with topotecan and cisplatin in recurrent/metastatic cervical cancer [11] and early trials in locally advanced disease [12]. A phase II randomized trial of cisplatin/RT with or without gemcitabine for locally advanced cervical cancer has found that the addition of gemcitabine increased the pathologic complete response rate compared to cisplatin/RT alone [13]. A phase III randomized trial of cisplatin/RT with or without concurrent gemcitabine and adjuvant cisplatin/gemcitabine found a significantly increased 3-year progression free survival, overall survival, and time to progression, favoring the combined chemotherapy regimen [14]. However, acute toxicity was significant, with 87% of patients experiencing grade ≥ 3 toxicity, compared to 46% in the standard arm. Notably, in the phase II randomized trial [13], grade ≥ 3 hematologic toxicity was also higher with gemcitabine (60% vs. 18%, $p<.05$), as was grade ≥ 2 gastrointestinal (GI) toxicity (60% vs. 40%, $p<.05$). These results indicate that intensive chemoradiation has considerable activity against cervical cancer, however, it is quite toxic. Methods to reduce toxicity during chemoradiotherapy, particularly gastrointestinal and hematologic, could mitigate this toxicity and take advantage of the therapeutic benefits of intensive concurrent chemotherapy.

4.3 Intensity Modulated Radiation Therapy

Conventional pelvic RT involves opposed anterior-posterior / posterior-anterior (AP/PA) and lateral fields, resulting in a box-shaped dose distribution that encompasses both targeted tissues (e.g. tumor, parametria, pelvic lymph nodes, etc.) and normal tissues (e.g., bowel, rectum, bladder, bone marrow, etc.). Field borders are typically defined based on standard bone landmarks rather than by expressly defined targets.

Intensity modulated radiation therapy (IMRT) is a modern RT technique that differs from conventional techniques in many ways. First, patients undergo computed tomography (CT) simulation so that customized target volumes can be defined 3-dimensionally. IMRT treatment planning involves multiple beam angles and uses computerized inverse

treatment planning optimization algorithms to identify dose distributions and intensity patterns that conform dose to the target, reducing radiation dose to surrounding tissues. IMRT delivery is typically accomplished with the use of multileaf collimators, which involve small motorized leaflets (collimators) that move in and out of the beam path, modulating the dose intensity.

Multiple studies in gynecologic cancer have shown that IMRT plans reduce dose to pelvic organs while maintaining acceptable target coverage [15-17]. Comparisons of IMRT to conventional treatments in patients have found reduced acute and late GI toxicity and hematologic toxicity with IMRT [18-20]. Studies have also shown that IMRT plans can be optimized to intensively reduce normal tissue dose [15-17] and have established evidence-based guidelines for dosimetric planning to reduce toxicity [21-23]. Predictive models indicate that optimized IMRT plans are clinically feasible and can be expected to decrease the rates of acute toxicity by approximately two-fold [21,23]. Recent retrospective studies have also indicated that IMRT is associated with low acute and late toxicity and favorable outcomes [24-26]. Prospective clinical trials of IMRT, however, are still limited. A prospective trial of IMRT for gynecologic cancer has shown IMRT to be feasible in the multi-institutional setting for post-operative patients [27], with low toxicity. A randomized trial of IMRT is ongoing in India [28]. This would be the first international randomized trial to test IMRT, with centralized quality assurance, for both postoperative and definitive treatment of cervical cancer.

4.4 Quality of Life and Patient-Reported Outcomes

A unique opportunity this study affords is to acquire prospective quality of life (QOL) data from a large international clinical trial. Chemoradiotherapy for cervical cancer is associated with significant acute and late toxicity, with corresponding reductions in QOL [29]. Validated QOL measurements used in patients treated for cervical cancer have demonstrated that a considerable proportion of patients report debilitating functional compromise and psychosocial morbidity [29]. Recent studies have indicated that reducing pelvic radiation dose in women with endometrial cancer reduces urinary incontinence, diarrhea, and fecal leakage, leading to fewer limitations in daily activities [30-31]. To our knowledge, however, detailed QOL data for cervical cancer patients treated with IMRT + cisplatin is lacking, particularly in the international setting. This trial provides an ideal mechanism to collect and analyze cross-cultural patient-reported longitudinal data. In this context, the question we will ask is whether IMRT is associated with superior acute and late QOL. Longitudinal assessments of QOL and key functions such as activities of daily living, bowel and urinary habits, psychosocial function, and sexual function (see Appendix IV) are included as secondary end points of this study.

We will explore the impact of IMRT on QOL using two validated instruments: the European Organization for Research and Treatment of Cancer (EORTC) QLQ-C30 form and QLQ-CX24 module. The 30-item EORTC QOL questionnaire (QLQC30) is a psychometrically robust, cross-culturally accepted questionnaire that was designed to be applicable to a broad spectrum of cancer patients as a core questionnaire [32]. The form has been translated into many languages including English, Mandarin, Hindi, Thai, Portuguese, Czech, Dutch, Korean, Spanish, Turkish, and Vietnamese. Previous studies have shown that, despite regional variations in responses that need to be considered, the QLQ-C30 is suitable for use in a wide variety of countries and settings [33].

The QLQ-CX24 is a validated and reliable psychometric module consisting of 3 multi-item scales and 5 single-item scales. It is characterized by high internal consistency,

good compliance and fast completion with no or minimal assistance [34]. Cross-cultural studies in Asian samples, for example, have found the module to be a reliable and valid measure of QOL [35-36]. The module has been translated into many languages including English, Mandarin, Hindi, Portuguese, Czech, Dutch, Korean, and Spanish.

4.5 Image-Guided Bone Marrow-Sparing IMRT

Hematologic toxicity (HT) is a key barrier to intensifying chemoradiotherapy in patients with pelvic malignancies. It is well-known that both radiation and chemotherapy are myelosuppressive, but the extent to which pelvic radiation contributes to HT in patients undergoing CRT is unknown. Radiation causes apoptosis of bone marrow (BM) and peripheral blood stem cells and BM stromal damage, resulting in myelosuppression and characteristic pathologic and radiographic BM changes [37-39]. BM stem cells are exquisitely sensitive to low doses of radiation [40]. Clinical studies have shown that radiation BM injury depends on both radiation dose and volume of BM irradiated [41-43]. CT-based IMRT plans can be optimized to reduce BM irradiation [16-17], but the large avoidance volume constrains IMRT plan optimization. Refining IMRT plans to focus on sparing BM subregions may therefore be a more effective strategy. Previous studies have shown that functional imaging can help optimize IMRT plans to spare active BM [44-47]. However, the locations of BM subregions most important for sparing remain unknown, and studies investigating functional BM imaging are needed to optimally design BM-sparing IMRT plans.

We have implemented two approaches to identifying key subregions of BM: (1) a “top-down” approach, using deformable image registration and high dimensional data analysis to statistically map BM subregions in which radiation dose is most likely to increase toxicity [47], and (2) a “bottom-up” approach, using functional imaging using PET and quantitative MRI to map functional BM [46]. With the “top-down” approach, BM volumes are deformed and dose is re-mapped to a common template using optical flow-based deformable registration. The dose vector for each patient is sampled from left to right, anterior to posterior, and from superior to inferior, resulting in a high resolution (3x3x2.5 mm^3) data matrix ("dose array"), with each row corresponding to a BM dose voxel in each patient. The position of each element of the array can be traced to its 3D location, preserving spatial information. We apply principal components analysis to reduce the data space to a lower dimensional space spanned by "eigendose" vectors, i.e. the eigenvectors of the dose array. The original dose vector is projected into this eigenspace, resulting in a set of principal component scores (PCs), which express that subject’s dose in terms of the new eigenbasis. We regress the toxicity value (e.g., white blood cell count nadir) on the top PCs, and from this regression model can derive weights that assign an importance level to each voxel, defining “critical” BM subregions. IMRT plans could be designed to avoid “critical” BM so-defined. However, validation studies testing this model in large data sets are needed to provide a rationale for this approach.

Alternatively, with a “bottom-up” approach, functional imaging is used to identify “active” BM subregions for avoidance. It is known from pathology and imaging studies that BM is comprised of subregions of hematopoeitically active, fat-poor, “red” BM and inactive, fat-rich, “yellow” marrow [48-52]. Red BM contains approximately 40% fat, 40% water, and 20% protein; yellow BM contains approximately 80% fat, 15% water, and 5% protein [48]. Approximately 50% of the body’s red BM is located in the pelvis and lumbar spine [53] and is thus contained within conventional RT ports. These active and inactive regions cannot be distinguished on computed tomography (CT) [54-55], which is the principal imaging modality used for RT planning. Functional imaging studies indicate that active BM tends to be concentrated particularly in vertebral and ilial subregions [44-

45,49]. These regions co-localize with regions which, by MRI studies, show longer T1 relaxation times and longer T2 indicative of red vs. yellow BM [38,48]. Reducing dose specifically to active subregions of pelvic BM may be beneficial, but the precise location of active BM subregions, variation in active BM distribution between individuals, and relationship between toxicity and radiation dose to these active BM regions are all unknown.

Qualitative MR has long been used to detect fat in tissues from the change in signal intensity caused by the characteristic short T1 of fat or the chemical shift difference between fat and water. Within BM there is considerable heterogeneity in fat content that varies with age and disease status. A quantitative technique called Iterative Decomposition of water and fat with Echo Asymmetry and Least-Squares Estimation (T2*-IDEAL) [56-59], can distinguish red and yellow BM based on their fat signal fraction, providing a quantitative assessment of BM in response to treatments. [18F]-deoxyfluorothymidine (^{18}F-FLT) is an effective PET imaging tracer for proliferating BM. FLT is a DNA precursor that is phosphorylated and sequestered intracellularly by the enzyme thymidine kinase 1 [60], which is active during DNA synthesis, leading to specific tracer uptake in proliferating tissues [61]. Hayman et al. recently showed was useful for *in vivo* measurements of proliferating BM [62]. Several studies have noted decreased tracer uptake in BM after irradiation with doses as low as 2 Gy, and complete absence of uptake after 10-20 Gy [61,63-65]. ^{18}F-FLT appears superior to ^{18}F-FDG for BM imaging, due to the inability of ^{18}F-FDG to discriminate between proliferating cells and metabolically active non-proliferating cells [62,66-67]. Recently a study found that increased radiation to BM sub-regions with higher ^{18}F-FDG-PET activity was associated with increased HT [68], supporting the hypothesis that reducing dose to active subregions could mitigate HT. In a sub-study of this protocol, we will test the hypothesis that BM-sparing IMRT plans designed to avoid active subregions identified by PET can further reduce HT. This technique could improve patients' tolerance to chemotherapy and increase the therapeutic ratio of CRT for pelvic malignancies in general.

4.6 Target Motion and Planning Margins

With CT-based treatment planning, a clinical target volume (CTV) is explicitly delineated on the planning CT. To account for variation in target position due to daily setup and organ and tumor motion, a planning margin is typically applied to the CTV, generating a planning target volume (PTV). Larger planning margins encompass increased amounts of normal tissue, limiting the ability of conformal techniques to reduce toxicity [69]. Precise knowledge of the size of planning margins required is necessary to optimize 3-D planning techniques, but evidence-based guidelines for PTV definition are presently lacking. Existing guidelines are largely based on historical norms and data from a limited number of small studies [70-72]. Larger studies to determine planning margins for cervix cancer are needed.

Recently, gantry-mounted kV cone beam computed tomography (CBCT) has been used to acquire daily volumetric images of patients in the treatment position [73]. The target volume can be drawn on the daily CBCT and the CBCT registered to the planning CT to determine the daily target position relative to planning CT. The margin that would be needed to encompass the target each day for a given degree of accuracy in target coverage can then be determined [73]. Using a surface landmarking protocol, we can also determine optimal asymmetric planning margins, by comparing the lengths of normals from the planning CTV surface to the CBCT surface at each landmark. Preliminary results indicate this approach could significantly reduce the size of the planning target volume (PTV) for the same probability of target coverage, which would reduce normal tissue irradiation [74]. This approach can also be used to generate a

shape model of organ motion, by identifying the major modes of variation in the target position [75]. Validation of the shape model and predicted margins in an independent and prospectively acquired data set is important prior to clinical implementation of this treatment planning approach.

4.7 Primary Hypothesis
Compared to conventional RT techniques, IMRT will reduce acute hematologic and gastrointestinal toxicity for cervical cancer patients treated with concurrent cisplatin.

5.0 OBJECTIVES
5.1 Primary
5.1.1 To test whether IMRT will reduce the rate of acute grade ≥ 3 hematologic or clinically significant grade ≥ 2 gastrointestinal toxicity (see section 16.1) compared to conventional RT techniques for cervical cancer patients treated with concurrent cisplatin.

5.2 Secondary
5.2.1 To determine the feasibility and quality assurance of IMRT for cervical cancer in an international cooperative group setting
5.2.2 To estimate and compare the probability of acute and late adverse events and the completeness of chemotherapy delivery with cisplatin/IMRT versus cisplatin/conventional RT
5.2.3 To estimate and compare efficacy of cisplatin/IMRT in terms of locoregional failure, disease-specific survival, disease-free survival, and overall survival.
5.2.4 To estimate and compare acute and long-term QOL associated with cisplatin/IMRT
5.2.5 To test the validity of a high-dimensional model of acute hematologic toxicity as a function of bone marrow dose distribution (substudy 1)
5.2.6 To quantify required planning margins based on analysis of daily kV cone beam computed tomography (substudy 2)
5.2.7 To quantify acute hematologic toxicity in subjects treated with functional bone marrow-sparing IMRT (substudy 3)

6.0 POPULATION AND ELIGIBILITY CRITERIA
6.1 Conditions for Patient Eligibility
6.1.1 Biopsy-proven, invasive squamous cell carcinoma, adenocarcinoma, or adenosquamous carcinoma of the cervix (clear cell and small cell neuroendocrine are not permitted)
6.1.2 Biopsy result positive for carcinoma within 60 days prior to registration
6.1.3 FIGO clinical stage I-IVA disease (see Appendix II), based on standard diagnostic workup, including:

- History/physical examination
- Examination under anesthesia (if indicated)

6.1.3.1 If the patient is status post hysterectomy, one or more of the following conditions must be present: positive lymph nodes, positive margins, parametrial invasion, or non-radical surgery (i.e., simple hysterectomy).
6.1.3.2 If the patient is inoperable, one or more of the following conditions must be present: clinical stage IB2-IVA, positive lymph nodes on nodal sampling or frozen section, and/or parametrial invasion

6.1.4 History/physical examination within 14 days prior to registration to document cervical tumor size and stage

6.1.5 Within 42 days prior to registration, the patient must have any of the following, if clinically indicated: examination under anesthesia, cystoscopy, sigmoidoscopy, rigid proctoscopy, or colonoscopy.
6.1.6 X-ray (PA and lateral), CT scan, or PET/CT scan of the chest within 42 days prior to registration;
6.1.7 CT scan, MRI, or PET/CT of the pelvis within 42 days prior to registration;
6.1.8 Karnofsky Performance Status 60-100 (see Appendix III)
6.1.9 Age ≥ 18
6.1.10 Laboratory data obtained ≤ 14 days prior to registration on study, with adequate bone marrow, hepatic and renal function defined as follows:

- Absolute neutrophil count (ANC) ≥ 1500 cells/mm^3;
- Platelets ≥ 100,000 cells/mm^3;
- Hemoglobin ≥ 10.0 g/dl (Note: The use of transfusion or other intervention to achieve Hgb ≥ 10.0 g/dl is acceptable)
- Creatinine clearance ≥ 50 mg/dl OR Serum Creatinine ≤ 1.5 x ULN
- Bilirubin < 1.5 mg/dl
- WBC ≥ 3,000/μl
- ALT/AST < 3 x ULN
-
- Negative serum pregnancy test for women of child-bearing potential

6.1.11 Women of childbearing potential must have a negative serum pregnancy test and must agree to practice effective birth control throughout their participation in the treatment phase of the study.
6.1.12 If there is clinical suspicion of AIDS, an HIV test must be done within 42 days prior to registration. Note: HIV positive patients with a $CD4^+$ T cell count > 200 per μL of blood and >14% of all lymphocytes are eligible for this trial.
6.1.13 Patients must sign informed consent prior to study entry.

6.2 Conditions for Patient Ineligibility

6.2.1 Prior invasive malignancy (except non-melanomatous skin cancer), unless disease free for a minimum of 3 years;
6.2.2 Prior systemic chemotherapy within the past three years
6.2.3 Prior radiotherapy to the pelvis or abdomen that would result in overlap of radiation therapy fields;
6.2.4 Para-aortic, inguinal, or gross (unresected) pelvic nodal metastasis. Gross pelvic nodal metastasis is defined as either:

- Radiographic evidence of nodal metastasis on CT or MRI (node having short axis diameter > 1 cm)
- Radiographic evidence of nodal metastasis on diagnostic FDG-PET or PET/CT scan (abnormally increased FDG uptake as determined and documented by the radiologist)
- Biopsy-proven metastasis (e.g. needle biopsy) in undissected node

6.2.5 Distant metastasis
6.2.6 Severe, active co-morbidity, defined as follows:

6.2.6.1 Unstable angina and/or congestive heart failure requiring hospitalization within the past 6 months;
6.2.6.2 Transmural myocardial infarction within the last 6 months;
6.2.6.3 Acute bacterial or fungal infection requiring intravenous antibiotics at the time of registration;

6.2.6.4 Chronic Obstructive Pulmonary Disease exacerbation or other respiratory illness requiring hospitalization or precluding study therapy at the time of registration;
6.2.6.5 Hepatic insufficiency resulting in clinical jaundice and/or coagulation defects;
6.2.6.6 Uncontrolled diabetes, defined as diabetes mellitus, which in the opinion of any of the patient’s physicians requires an immediate change in management; a patient may be considered eligible for the study if the physician managing the patient’s diabetes considers that the appropriate changes in management have resulted in adequate control.
6.2.6.7 Uncompensated heart disease or uncontrolled high blood pressure, which in the opinion of any of patient’s physicians, requires immediate change in management; a patient may be considered eligible for the study if the physician managing the patient’s heart disease or blood pressure considers that the appropriate changes in management have resulted in adequate control.
6.2.6.8 Acquired Immune Deficiency Syndrome (AIDS) based upon current CDC definition; patients with AIDS will be ineligible for this protocol because the treatments involved may be significantly immunosuppressive. Patients with clinical suspicion of AIDS and who are unwilling to have an HIV test are not eligible for this trial.
6.2.6.9 Uncontrolled infection
6.2.6.10 Other immunocompromised status (e.g., organ transplant or chronic glucocorticoid use).

6.2.7 Women who are pregnant or lactating are ineligible due to teratogenic effects on developing fetuses. Women who are of child-bearing potential need to practice effective methods of contraception including oral contraceptives, intrauterine device, diaphragm with spermicides, and/or abstinence.
6.2.8 Women participating on substudy 2 or substudy 3 should not have a prior history of hip, pelvic, or lumbosacral prosthesis or other implanted device.

7.0 PRE-TREATMENT EVALUATIONS / MANAGEMENT

7.1 Additional Mandatory Pre-treatment Evaluations/Interventions

7.1.1 History and physical examination including assessment of tumor size, height, weight and body surface area and performance status . Initial examination should be performed by both a gynecologic oncologist and a radiation oncologist, either independently or jointly, with at least one examination occurring within 14 days prior to study entry
7.1.2 Assessment of smoking and alcohol history (past and current) within 14 days prior to study entry.
7.1.3 Assessment of race and ethnicity and comorbidity index within 14 days prior to study entry
7.1.4 Patients must not be allergic to iodinated contrast if undergoing a contrast enhanced CT scan of the pelvis.
7.1.5 Pre-treatment PET and MRI are not required but are encouraged to aid staging evaluation and target delineation. Analyses of toxicity and outcomes will be stratified according to the use of pre-treatment image-guidance.

8.0 REGISTRATION PROCEDURES

8.1 Pre-Registration Requirements

8.1.1 Pre-Registration Requirements for IMRT

In order to register patients on this study, the institution must provide baseline physics information and an anonymized case study, including all contours and a sample treatment plan ("Dry Run"). Instructions for completing these requirements are available at the International Radiotherapy Technologies and Oncology Consortium (IRTOC) website: http://radonc.ucsd.edu/research/irtoc/Pages/data-transfer.aspx

To pre-register, each institution should do the following:

- Contact irtoc@ucsd.edu to request an FTP account for digital data submission
- Submit a Physics Questionnaire Form (PQF) (see Appendix V) to UC San Diego
- Contact the Washington University Image-Guided Therapy QA Center (ITC) itc@castor.wustl.edu to request an FTP account for digital data submission
- Transmit "Dry Run" data to the ITC

Instructions for data transfer to UC San Diego can be found at website: http://radonc.ucsd.edu/research/irtoc/Pages/data-transfer.aspx

Instructions for data transfer to the ITC can be found at http://atc.wustl.edu/protocols/index.html

An IMRT phantom study may be required to be eligible to register patients. Institutions will be notified with further instructions if they are required to submit a phantom study. Instructions for requesting and irradiating the phantom will be available at the Radiological Physics Center (RPC) at MD Anderson Cancer Center website (http://rpc.mdanderson.org/rpc)

8.1.2 IMRT Plan Central Review

Dry Run data will be reviewed centrally at UC San Diego. An assessment about protocol compliance for the Dry Run will be made and communicated to the participating institution. An additional case study may be required if the initial review reveals protocol non-compliance.

IMRT plans for all patients enrolled on the study will be centrally reviewed to determine protocol compliance (**see section 9.5.1**). IMRT data submission is encouraged, but not required, *prior* to the patient's start date, to provide the opportunity for pre-treatment planning modifications.

8.1.3 "Wet Run" Quality Control

For each center, after the first subject is enrolled, registration will be suspended in order to permit central quality control analysis on the first plan submission. If planning deviations are identified, the center will be notified so that the deviations may be corrected, if possible. This process of staggered enrollment will be repeated for subsequent subjects until a subject is enrolled whose first IMRT plan submission meets protocol requirements. After this condition is met, enrollment may proceed without staggering.

8.2 Registration

8.2.1 Screening Procedures

Diagnostic or laboratory studies will be performed to determine eligibility. Studies or procedures that were performed for clinical indications or as standard of care (not

exclusively to determine study eligibility) may be used for baseline values even if the studies were done before informed consent was obtained.

8.2.2 Informed Consent

Written informed consent will be obtained prior to any study procedures (see Appendix I). Each institution should customize the consent form according to their institutional requirements. The investigational nature and objectives of the trial, the procedures and treatments involved and their attendant risks and discomforts, and potential alternative therapies will be carefully explained to the subject and a signed informed consent will be obtained. Documentation of informed consent (e.g. scanned record) should be transmitted to UC San Diego via the secure FTP server.

8.2.3 Recruitment

Subjects will be recruited at participating centers by local investigators.

8.2.4 Registration Procedures

Patients can be registered by emailing irtoc@ucsd.edu or through the IRTOC website at http://radonc.ucsd.edu/irtoc/intertecc/webregistration.asp. A UCSD login and password are required to use the web registration. Once UC San Diego's coordinator has verified that the patient is eligible (via transmitted eligibility check-list), has signed informed consent, and that the institution has met regulatory requirements, the patient is assigned a case number. UC San Diego's coordinator will send the institution a data submission calendar including all data forms, images, and reports and the dates on which they are due.

8.2.5 Accrual cap

Accrual for the phase II and phase III portions of the study will be capped at 20 and 75 patients per institution, respectively. If either component accrues poorly as a result of the cap a protocol amendment may be submitted to abolish the cap. Accrual cap does not apply to institutions participating in substudies 2 or 3.

9.0 RADIATION THERAPY

9.1 General

The total duration of chemotherapy and radiotherapy should be ≤ 60 days. For post-operative patients, chemoradiotherapy should be initiated within 8 weeks following surgery.

9.2 External Beam Radiation Therapy

9.2.1 Simulation

9.2.1.1 Bladder and Bowel Preparation

The degree of bladder and rectal fullness should be made to duplicate that which is anticipated for daily treatment, i.e., if the patient is instructed to maintain a full bladder for treatment, she should be simulated as such. It is recommended to use a consistent bladder filling state (e.g. always full or always empty) for simulation and treatment. It is recommended for patients not to be simulated or treated with a full rectum as this may result in irreproducible setup. Bowel preparatory agents (enema, stool softeners, etc.) may be applied at the discretion of the physician. If a full rectum is noted on the simulation study it is recommended that the patient undergo a new simulation scan.

9.2.1.2 Contrast and Markers

Intravenous contrast is recommended unless medically contraindicated. Oral contrast is optional. A radio-opaque cervical marker may be placed in the apex of the vagina to assist with target delineation and is optional. Implanted fiducials are optional.

9.2.1.3 Position
Simulation will be done in the supine position.

9.2.1.4 Immobilization
All subjects will have a customized immobilization device (e.g., Alpha Cradle or Vac-Lok) fabricated at the time of simulation.

9.2.1.5 Imaging
All subjects will undergo a CT (or PET/CT) simulation scan using a slice thickness of 2.5-5.0 mm (preferably ≤ 3.0 mm) and large field-of-view pelvic protocol. Patients on the kV CBCT substudy must have a simulation CT with slice thickness ≤ 3.0 mm (see section 9.3). CT scans will be obtained from the T12 vertebral body to 5 cm below the ischial tuberosities.

9.2.1.6 Isocenter placement
Isocenter placement is left to discretion of treating physician except in patients undergoing CBCT with each fraction (see section 9.3). For patients undergoing CBCT with each fraction, the isocenter should be placed along the patient's midline 1.5 cm caudal to the inferior border of the sacroiliac joint.

9.2.2 Target Delineation
9.2.2.1 General
Pelvic MRI and/or PET fusion with the simulation scan is recommended to aid target delineation. Fusion should be optimized to match the MRI / PET scan to the treatment position. The Gross Tumor Volume (GTV) and Clinical Target Volume (CTV) and normal tissues will be contoured on all CT slices in which the structures exist. The definition of all volumes will be in accordance with the 1993 ICRU Report #50: Prescribing, Recording and Reporting Photon Beam Therapy (http://www.icru.org/index.php?option=com_content&task=view&id=72).

9.2.2.2 GTV Delineation
The GTV is defined as all known gross disease determined from radiographic studies, clinical information, physical examination, endoscopic examination, and biopsy results. If the GTV is not readily identifiable, it is acceptable to omit GTV delineation, since this is not required for treatment planning.

9.2.2.3 CTV Delineation
The CTV is defined as the gross tumor plus areas containing potential microscopic disease, including the cervix, uterus (if present), the superior third of the vagina (or half of the vagina, if clinically involved), the parametria, and the regional lymph nodes. CTV delineation should follow reported consensus guidelines [76-78]. A visual demonstration of target delineation procedure can be downloaded from our website at http://radonc.ucsd.edu/irtoc/intertecc/videos.asp

It is suggested to divide the CTV into 3 subregions: CTV1, CTV2, and CTV3.

CTV1 will consist of the gross tumor, cervix, and uterus (if present). In postoperative patients, CTV1 should be drawn as the vaginal cuff and include 3 cm of the proximal vagina. CTV2 will consist of the parametria and superior third of the vagina (or half of the vagina, if clinically involved). CTV3 will include the common, external, and internal iliac and presacral lymph nodes. The upper border of the CTV3 should not extend above the confluence of the common iliac arteries with the aorta (i.e., aortic bifurcation), and should begin no lower than the level of the inferior border of the L4-5 interspace. The nodal CTV (CTV3) will be obtained by applying a 7 mm margin around the vessels, plus extension to include any adjacent visible lymph nodes, lymphoceles, or pertinent surgical clips [76]. The presacral nodes should be contoured until the superior border of the S3 vertebral body is reached; below this point the nodal volume can be separated into two structures. The external iliac nodes should be contoured to the superior aspect of the femoral head. CTV3 will be modified to exclude bone, muscle, and bowel, per consensus guidelines [76]. The CTV should not extend inferior to the ischial tuberosities.

For patients with distal one third vaginal involvement, the inguinal nodes will be contoured continuously from the external iliac nodes to 2 cm caudad to the saphenous/femoral junction. For contouring guidelines for inguinal nodes, refer to atlases used to treat anal carcinoma [79-80].

9.2.2.4 PTV Delineation

The Planning Target Volume (PTV) will add a margin around CTV1-3 to compensate for treatment setup and internal organ motion. Around CTV1, a 15 mm uniform expansion should be used. Around CTV2, a 10 mm uniform expansion should be used. Around CTV3, a 7 mm uniform expansion should be used. These expansions will generate PTV1, PTV2, and PTV3, respectively. PTV1-3 will be fused to generate the PTV. The PTV should be manually or automatically trimmed up to 3 mm from the skin surface, if necessary, to spare skin, provided that the CTV is still included entirely within the PTV. No other modifications to the PTV are allowed except as described in section 9.2.2.5.

9.2.2.5 Internal Target Volume (ITV)

Use of an ITV is strongly encouraged if daily volumetric IGRT will not be used. In this case, patients should be simulated with both a full and empty bladder (i.e., 2 simulation scans). CTV1-CTV3 and PTV1-PTV3 should be delineated as described above on the plan used for treatment (either the full or empty bladder scan), and CTV1 should be delineated on both scans. The CTV1 from both scans should be fused together to generate the ITV. A 7 mm margin should be applied to generate PTV4. PTV4 should be fused with PTV1-PTV3 to generate the final PTV. A visual demonstration of this procedure can be downloaded from our website at http://radonc.ucsd.edu/irtoc/intertecc/videos.asp.

9.2.3 Normal Tissue Delineation

9.2.3.1 General

Normal tissues will be contoured on the simulation scan. The tissue within the skin surface and outside all other critical normal structures and the PTV is designated as unspecified tissue. Critical normal tissues for IMRT optimization will consist of the bowel, bladder, rectum, and bone marrow. Femoral heads will be contoured to determine delivered dose but will not be used as a planning

constraint.

9.2.3.2 Bowel
The bowel will be contoured beginning from the axial slice situated 1 cm superior to the superior-most slice containing PTV (if bowel is not present at this level, the bowel contour will start from its most superior extent), and will continue to its most inferior extent in the pelvis. The outermost extent of the bowel loops will be outlined on each axial CT slice, as described in the literature [22]. Individual loops of bowel should not be contoured separately. Rectum should be contoured separately from bowel (section 9.2.3.3). An instructional video can be downloaded from http://radonc.ucsd.edu/irtoc/intertecc/videos.asp

9.2.3.3 Rectum
The outer rectal wall will be contoured and filled in, treating the organ as a solid continuous structure, and will be defined from the level of the sigmoid flexure to the anus.

9.2.3.4 Bladder
The outer bladder wall will be contoured and filled in, treating the organ as a solid continuous structure.

9.2.3.5 Bone Marrow
The outer bone contour will be delineated and filled in, treating the bone marrow as a solid continuous structure. The regions contoured will include the os coxae, L5 vertebral body, entire sacrum, acetabulae, and proximal femora. The superior extent of the bone marrow contour should be at the level of the superior border of L5 or the iliac crest, whichever is more superior. The caudal-most extent of the bone marrow contour should be at the level of the ischial tuberosities. For examples see Mell et al. [22], Figure 3. An instructional video can be downloaded from http://radonc.ucsd.edu/irtoc/intertecc/videos.asp

9.2.3.6 Femoral Heads
The outer contours of the femoral heads will be delineated and filled in, treating each as a solid continuous structure. Do not include the femoral neck.

9.2.3.7 Functional Bone Marrow (Substudy 3 only)
Functional BM will be a subset of the entire BM volume (delineated in 9.2.3.5). Functional BM will be defined as the subregion with a standardized uptake value (SUV) greater than the mean value over the BM volume. Automatic segmentation using commercially available software can be used to define the functional BM volume, which will be used as an avoidance structure for IMRT planning. The functional imaging technique will be either FDG-PET or FLT-PET depending on availability of funding for FLT-PET.

9.2.4 Treatment Planning

An instructional video on treatment planning and evaluation can be downloaded from http://radonc.ucsd.edu/irtoc/intertecc/videos.asp

9.2.4.1 Beam arrangement
Intensity-modulated radiation therapy plans may include static field arrangements

(e.g. 5-9 fields), modulated arc therapy, or Tomotherapy. Conventional RT will consist of a 4-field "box" arrangement using opposed AP/PA and lateral fields. For conventional RT, it is permissible to use bone landmarks to draw field borders or to use 3-D planning with explicit targeting as outlined above, using customized blocking to encompass the PTV. If explicit targeting is used, follow the target coverage requirements in 9.2.4.4. If bone landmarks are used, use the following portals:

- Superior border: L4-5
- Lateral border: 1-2 cm lateral to the border of the true pelvis
- Inferior border: Obturator foramen or 4 cm inferior to vaginal cuff, whichever is lower
- Anterior border: line from pubic symphysis to 1 cm anterior to common iliac nodes at L4-5
- Posterior border: draw border posterior to or splitting the sacrum from S1-S4
- Custom blocking to shield femoral heads. Do not block the obturator foramen or within 1 cm of the common iliac nodes

.

9.2.4.2 Photon energy
IMRT plans will use 6-15 MV photons. Conventional plans may use 6-18 MV photons.

9.2.4.3 Prescription dose
The prescription dose for patients with intact cervix cancer will be 45.0 Gy in 1.8 Gy fractions to the PTV. The prescription dose for post-operative patients will be 50.4 Gy in 1.8 Gy fractions to the PTV.

9.2.4.4 Target coverage requirements

- The 99% prescription isodose surface will encompass ≥ 90% of the PTV.
- ≥ 99% of the PTV will receive ≥ 90% of the prescription dose.
- ≥ 97% of the PTV will receive ≥ 97% of the prescription dose.
- < 1% of PTV will receive ≥ 115% of the prescription dose.
- < 10% of the PTV will receive ≥ 110% of the prescription dose.
- Dose maximum should occur within the PTV

9.2.4.5 Normal tissue planning goals for IMRT (soft constraints):

- Bowel: volume receiving >45 Gy (V_{45}) ≤ 200 cc; V_{40} < 30%; maximum dose < 50 Gy
- Rectum: V_{45} < 50%; V_{30} < 60%; maximum dose < 50 Gy
- Bone Marrow: V_{10} < 80%; V_{20} < 66%
- Bladder: V_{45} < 50%; maximum dose < 50 Gy
- Femoral Head: V_{30} < 15%; maximum dose < 50 Gy
- Functional Bone Marrow (Substudy 3 only): mean dose < 15 Gy; V_{10} < 75%; V_{20} < 65%

9.2.4.6 Normal tissue requirements for IMRT (hard constraints):

- Bowel: volume receiving >45 Gy (V_{45}) ≤ 250 cc; maximum dose < 115%
- Rectum: maximum dose < 115%
- Bone Marrow: V_{10} < 90%; V_{20} < 75%

- Bladder: maximum dose < 115%
- Femoral Head: maximum dose < 115%
- Functional Bone Marrow (Substudy 3 only): mean dose < 25 Gy; V_{10} < 80%; V_{20} < 70%

9.2.4.7 Treatment Delivery
Treatment will be delivered on a linear accelerator; IMRT should be delivered using multi-leaf collimation. Patients will be aligned to skin marks prior to treatment and position will be verified using planar imaging as specified in section 9.2.6.

9.2.4.8 Parametrial Boost
Parametrial boost is discouraged unless considered mandatory in the best interest of the patient according to the discretion of the treating physician.
In this case either brachytherapy or external beam radiotherapy (e.g., opposed anterior-posterior field arrangements with appropriate blocking, to a total dose of 8.0-14.4 Gy in 4-8 daily fractions) is acceptable.

9.2.4.9 Re-planning
Re-planning (such as to account for changes in tumor volume) is allowed. If re-planning is necessary, the new treatment plan should meet the same criteria as the initial plan, as if the new plan were delivered for the entire treatment course (i.e., 25 or 28 fractions). The new treatment plan should be submitted for central review according to the same process as the initial plan.

9.2.4.10 Heterogeneity corrections
Heterogeneity corrections should be applied.

9.2.5 External Beam Treatment Modification
Treatment will be held for ANC <500/mm^3, platelets < 20,000 mm^3, febrile neutropenia, or uncontrolled bleeding. No dose reductions are allowed. Treatment will be resumed upon resolution of neutropenia (ANC ≥500/mm^3), thrombocytopenia (platelets ≥ 20,000 mm^3), and febrile neutropenia (temperature<38.0 ° C).

9.2.6 Setup Verification
Skeletal imaging (e.g., electronic portal MV or kV imaging or CBCT) should be performed at least weekly to verify setup accuracy. For patients treated with conventional techniques, MV portal verification is recommended at least weekly. Daily on-line imaging (image-guided radiotherapy (IGRT)) is allowed but not required. For patients undergoing daily kV CBCT, refer to specifications in section 9.3.

9.2.7 Radical IMRT for boost therapy
Use of IMRT in place of brachytherapy to boost gross pelvic disease is expressly disallowed and is considered off-protocol therapy. Use of IMRT in place of brachytherapy is strongly discouraged. If deemed necessary or essential for the patient's care (e.g., if a patient refuses brachytherapy), please notify the study PI. Patients treated in this fashion will be analyzed for outcomes according to intent-to-treat.

9.3 Cone Beam Computed Tomography

9.3.1 General

For patients undergoing CBCT with each fraction: At the time of simulation, the isocenter should be placed along the patient's midline 1.5 cm caudal to the inferior border of the sacroiliac joint. The CBCT will be used for data collection only and not for soft tissue alignment. If a marked discrepancy is observed between the patient's imaged anatomy on the day of treatment versus the day of simulation, at the treating physician's discretion, the patient may be taken down off the machine (e.g., to void the bladder or rectum) and may be treated later that same day. If the anatomical discrepancy is still present, the treating physician may elect to postpone treatment.

9.3.2 CBCT protocol

Patients will undergo CBCT acquisition prior to each fraction, for a total of 25 scans. CBCTs will be obtained in half-fan mode, using 125kV, 80mA, and 25ms/frame. Axial images of the pelvis will be acquired and DICOM files will be transmitted centrally for analysis after the conclusion of treatment, preferably within 2-3 weeks of completion.

9.4 Brachytherapy

9.4.1 General

Either low dose rate (LDR) or high dose rate (HDR) brachytherapy is permitted according to each institution's standard. Either standard (point-directed) or volume-directed brachytherapy techniques are permitted according to each institution's standard. Participating institutions will declare whether they intend to use LDR or HDR and point-dose or volume-directed brachytherapy prior to enrolling their first patient. For institutions wishing to implement pulse dose rate (PDR), please contact the study P.I. Institutions must adhere to their declared brachytherapy regimen for all patients treated on this protocol. If an institution intends to change its brachytherapy regimen, a request must be submitted in writing to IRTOC before enrolling additional patients. Interstitial brachytherapy may be used to treat disease that cannot be adequately treated with intracavitary treatment. Brachytherapy may be initiated no sooner than the fourth week of treatment. External beam radiation and brachytherapy may not be administered on the same day. It is recommended to start brachytherapy after completion of external beam RT. Initiation of brachytherapy must not occur before delivery of at least 39.6 Gy of external beam RT.

9.4.2 High dose rate, point-directed brachytherapy (intact cervix)

9.4.2.1 Imaging

Orthogonal x-rays, CT, and/or MRI may be used for brachytherapy planning

9.4.2.2 Prescription Dose and Fractionation

Dose will be prescribed to point A according to each institution's standard, provided normal tissue constraints in section 9.4.3.4 are met. Permissible dose/fractionation schemes are:

- 5.5 Gy x 6 fractions
- 5.5 Gy x 5 fractions
- 6.0 Gy x 5 fractions

- 6.0 Gy x 6 fractions
- 7.0 Gy x 3 fractions
- 7.0 Gy x 4 fractions
- 7.0 Gy x 5 fractions
- 7.5 Gy x 3 fractions

In general, insertions should be separated by a minimum of 48 hours and no more than 2 insertions should be performed per week. Please notify the study P.I. in advance if insertions within 48 hours of each other are planned or required.

9.4.2.3 Source
Iridium-192 is the source for HDR brachytherapy.

9.4.2.4 Normal Tissue Constraints
Maximum allowable cumulative doses (external beam + brachytherapy) to normal tissues are (in EQD2, α/β=3, $T_{1/2}$ = 1.5 hours): bladder (ICRU reference point), 80 Gy; rectum (ICRU reference point), 75 Gy. It is recommended that the rectum and bladder for each fraction receive less than or equal to 70% and 80% of the point A dose, respectively, if feasible. If CT is used for planning it is recommended to keep the maximum bowel dose < 25% of the brachytherapy prescription dose. Every attempt should be made to deliver the full prescription dose, even if the late responding tissues receive a slightly higher dose.

9.4.3 Low dose rate, point-directed brachytherapy (intact cervix)
9.4.3.1 Imaging
Orthogonal x-rays, CT, and/or MRI may be used for brachytherapy planning

9.4.3.2 Prescription Dose and Fractionation
35-40 Gy (80-85 Gy total in EQD2, α/β=3, $T_{1/2}$ = 1.5 hours) will be delivered to point A in 1-2 insertions. If 2 insertions are used they should be separated by a minimum of 7 and maximum of 21 days.

9.4.3.3 Source
Cesium-137 is the source for LDR brachytherapy.

9.4.3.4 Normal Tissue Constraints
Maximum allowable cumulative doses (external beam + brachytherapy) to normal tissues are (in EQD2, α/β=3, $T_{1/2}$ = 1.5 hours): bladder (ICRU reference point), 85 Gy; rectum (ICRU reference point), 80 Gy; vaginal surface (reference point), 135 Gy. If CT is used for planning it is recommended to keep the maximum bowel dose < 25% of the brachytherapy prescription dose. Every attempt should be made to deliver the full prescription dose, even if the late responding tissues receive a slightly higher dose.

9.4.4 High dose rate, volume-directed brachytherapy (intact cervix)
9.4.4.1 Imaging
Pelvic MRI (≤ 5 mm slice thickness) is required with either the first or second insertion. An MRI-compatible applicator is required to perform volume-directed brachytherapy. Subsequent insertions may use CT or MRI for planning.

9.4.4.2 Target and Normal Tissue Delineation
The following targets and organs at risk (OAR) will be contoured at each insertion using the T2 weighted (para)-transversal MRI sequences, according to the GEC ESTRO Recommendations [81-82]. The MRI based target delineation can be reused by superimposition in the process of contouring on CT, if for subsequent fractions of brachytherapy only CT can be used with the applicator in place. No planning margins will be added to the CTV.

- GTVB: Macroscopic tumor (if present) at time of brachytherapy
- High risk target (HR-CTV): GTVB + whole cervix + presumed extra cervical tumor extension
- Point-A (left and right)
- Bladder (the outer bladder wall is contoured)
- Rectum (the outer rectal wall is contoured from above the anal sphincter to the level of transition into the sigmoid)
- Sigmoid (the outer sigmoid wall is to be contoured from the recto-sigmoid flexure to 2 cm superior to the parametria and the uterus
- ICRU bladder point
- ICRU rectal point

9.4.4.3 Prescription Dose and Fractionation
Minimum allowable cumulative dose (external beam + brachytherapy) to HR-CTV D90 is 75 Gy (EQD2, $\alpha/\beta=3$, $T_{1/2} = 1.5$ hours) and maximum allowable cumulative dose (external beam + brachytherapy) to HR-CTV D90 is 90 Gy (EQD2). Insertions should be separated by a minimum of 48 hours and no more than 2 insertions should be performed per week.

9.4.4.4 Normal Tissue Constraints
Maximum allowable cumulative doses (external beam + brachytherapy) to normal tissues are (in EQD2, $\alpha/\beta=3$, $T_{1/2} = 1.5$ hours): bladder (ICRU reference point), 85 Gy; rectum (ICRU reference point), 80 Gy. If CT is used for planning it is recommended to keep the maximum bowel dose < 25% of the brachytherapy prescription dose. In addition, it is suggested to meet the following constraints (EQD2, $\alpha/\beta=3$, $T_{1/2} = 1.5$ hours):

- Rectum $D_{2cc} < 75\ Gy_{EQD2}$ (cumulative)
- Sigmoid $D_{2cc} < 75\ Gy_{EQD2}$ (cumulative)
- Bladder $D_{2cc} < 90\ Gy_{EQD2}$ (cumulative)

Doses should be converted to EQD2 doses by the following formula:

EQD2= D X [(d + α/β)/(2 + α/β)}. Every attempt should be made to deliver the full prescription dose, even if the late responding tissues receive a slightly higher dose.

9.4.5 Low dose rate, volume-directed brachytherapy (intact cervix)

9.4.5.1 Imaging
Identical to section 9.4.4.1.

9.4.5.2 Target Delineation
Identical to section 9.4.4.2.

9.4.5.3 Prescription Dose and Fractionation
The dose to 90% or more of the volume (D90) for the HR-CTV will be 35-40 Gy (EQD2, α/β=3, $T_{1/2}$ = 1.5 hours), delivered in 1-2 insertions. If 2 insertions are used they should be separated by a minimum of 7 and maximum of 21 days.

9.4.5.4 Normal Tissue Constraints
Identical to section 9.4.4.4

9.4.6 Post-operative brachytherapy

For post-operative patients it is permissible to deliver adjuvant brachytherapy to the vaginal cuff using a vaginal cylinder or ovoids. In this case the upper 3 cm of the vagina should be treated. Permissible dose / fractionation schemes are:

- 5.0 Gy x 2 fractions to 0.5 cm from the cylinder surface
- 5.5 Gy x 2 fractions to 0.5 cm from the cylinder surface
- 6.0 Gy x 2 fractions to 0.5 cm from the cylinder surface
- 6.0 Gy x 3 fractions to the vaginal surface
- 7.0 Gy x 3 fractions to the vaginal surface

9.4.7 Intracavitary Radiotherapy Dosimetry

9.4.7.1 The dose to points A and B, the rectal reference point dose, bladder reference point dose, and vaginal surface reference point dose, and central axis isodose curve must be calculated and reported.

9.4.7.2 Point A: Measure 2 cm along the intrauterine tandem from the cervical os or flange of the tandem and 2 cm laterally in the plane of the intracavitary system.

9.4.7.3 Point B: Measure 5 cm lateral from a point 2 cm vertically superior to the cervical os or flange of the central tandem along the patients' midline.

9.4.7.4 Bladder Dose: Calculated at the center (in the superior-inferior plane on AP view) of a contrast-filled balloon of a Foley catheter and closest to the applicator system on a lateral view, as defined by ICRU 38.

9.4.7.5 Rectal dose: In accordance with ICRU 38, mark the point 0.5 cm posterior to the vaginal surface (as demarcated by the opaque packing) at the midpoint of the applicator system or at the level of the flange if no ovoids are used.

9.4.7.6 Dose and volume reporting for volume-directed brachytherapy
Uniform dose volume reporting according to the GEC ESTRO guidelines [81-82]. is required. For each fraction the following parameters should be recorded:

- TRAK
- D100 for GTVB, HR-CTV
- D90 for GTVB, HR-CTV
- D50 for HR-CTV
- V100 for HR-CTV
- D_{2cc} of the bladder, rectum and sigmoid, (converted to EQD2 doses per formula $EQD2= D \times [(d + \alpha/\beta)/(2 + \alpha/\beta))$; use $\alpha/\beta=3$.
- ICRU bladder and ICRU rectal point

9.4.8 Instruments
Tandem and ring or tandem and ovoids will be used for intact cervix brachytherapy. A standard applicator, such as the Fletcher-Suit-Delclos applicator, is recommended. For patients with lower third vaginal involvement, tandem and cylinder may be most appropriate and may be used at the discretion of the treating radiation oncologist. Similarly, interstitial brachytherapy (such as with a Vienna applicator) is allowed at the discretion of the treating physician. A tandem and cylinder or tandem alone is allowed only for patients where tandem and ring or ovoid application is not possible due to extent of disease or poor anatomy (e.g., obliterated fornices). A vaginal cylinder or ovoids may be used to treat the vaginal cuff in post-hysterectomy patients with positive margins.

9.5 Protocol Compliance and Quality Assurance Review

9.5.1 Quality assurance to verify protocol compliance will be performed by the IMRT protocol review committee at the UC San Diego Center for Advanced Radiotherapy Technologies (CART). Simulation scans and treatment planning data will be transmitted to the ITC for central review. Instructions for data transfer to the ITC can be found at http://atc.wustl.edu/protocols/index.html.

Investigators are strongly encouraged to submit treatment plans *prior* to initiating treatment, so that feedback may be provided to ensure protocol compliance. Otherwise, data should be submitted as soon as possible following completion of the plan, and no later than 1 month following completion of treatment. Criteria for protocol compliance are listed below.

9.5.2 Simulation

9.5.2.1 Position

- Per Protocol: supine
- Unacceptable (Major) Deviation: not supine

9.5.2.2 CT Slice Thickness

- Per Protocol: ≥ 2.5 mm and ≤ 3.0 mm
- Minor Deviation: > 3.0 mm and ≤ 5.0 mm
- Unacceptable (Major) Deviation: < 2.5 mm or > 5.0 mm

9.5.2.3 CT Range
- Per Protocol: T12 – 5.0 cm inferior to ischial tuberosity
- Minor Deviation: ≤ 2 cm deviation from protocol
- Unacceptable (Major) Deviation: > 2 cm deviation from protocol

9.5.3 Target Delineation (IMRT or 3-D planning)

9.5.3.1 CTV1
- Per Protocol: includes gross tumor, cervix, and uterus as described in section 9.2.2.3
- Minor Deviation: ≤ 1 cm deviation from volumes as described in section 9.2.2.3
- Unacceptable (Major) Deviation: fails to include gross tumor, cervix, or uterus and/or any other deviation > 1 cm from volumes as stated in section 9.2.2.3

9.5.3.2 CTV2
- Per Protocol: includes parametria and superior one third to one half of vagina as described in section 9.2.2.3
- Minor Deviation: ≤ 1 cm deviation from volumes as described in section 9.2.2.3
- Unacceptable (Major) Deviation: fails to include parametria or superior vagina and/or any other deviation > 1 cm from volumes as stated in section 9.2.2.3

9.5.3.3 CTV3
- Per Protocol: includes common, external iliac, internal iliac, and presacral lymph nodes as described in section 9.2.2.3
- Minor Deviation: ≤ 1 cm deviation from volumes as stated in section 9.2.2.3
- Unacceptable (Major) Deviation: upper border extends superior to aortic bifurcation or inferior to lower border of L4/5 interspace, and/or lower border extends inferior to ischial tuberosity, and/or any other deviation > 1 cm from volumes as stated in section 9.2.2.3

9.5.4 PTV Delineation (IMRT or 3-D planning)

9.5.4.1 PTV1
- Per Protocol: 15 mm margin on CTV1
- Minor Deviation: up to 20 mm margin on CTV1
- Unacceptable (Major) Deviation: < 15 mm or >20 mm margin on CTV1

9.5.4.2 PTV2

- Per Protocol: 10 mm margin on CTV2
- Minor Deviation: up to 15 mm margin on CTV2
- Unacceptable (Major) Deviation: < 10 mm or >15 mm margin on CTV1

9.5.4.3 PTV3

- Per Protocol: 7 mm margin on CTV3
- Minor Deviation: 5-7 mm or 7-10 mm margin on CTV3
- Unacceptable (Major) Deviation: < 5 mm or >10 mm margin on CTV3

9.5.5 Normal Tissue Delineation (IMRT or 3-D planning)

9.5.5.1 Bowel, Rectum, Bladder, and Femoral Heads

- Per Protocol: according to sections 9.2.3.2-9.2.3.4 and 9.2.3.6
- Minor Deviation: ≤ 1 cm deviation from volumes as described in section 9.2.3.2-9.2.3.4 and 9.2.3.6
- Unacceptable (Major) Deviation: > 1 cm from volumes as stated in section 9.2.3.2-9.2.3.4 and 9.2.3.6

9.5.5.2 Bone Marrow

- Per Protocol: according to section 9.2.3.5
- Unacceptable (Major) Deviation: not per section 9.2.3.5

9.5.5 Field Borders (Conventional RT)

- Per Protocol: according to section 9.2.4.1
- Minor Deviation: ≤ 1 cm deviation from borders as stated in section 9.2.4.1
- Unacceptable (Major) Deviation: > 1 cm deviation from borders as stated in section 9.2.4.1

9.5.6 Target Dose

- Per Protocol: according to guidelines as stated in sections 9.2.4.3, 9.2.4.4, 9.4.2.2, 9.4.3.2, 9.4.4.3, 9.4.5.3, and 9.4.6
- Minor Deviations:

  Prescription Dose (Intact): 40.0-44.9 or 45.1-50.4 Gy
  Prescription Dose (Postoperative): 45.0-50.3 or 50.5-54.0 Gy
  99% isodose: encompasses 85-90% of the PTV
  Dose to 99% of PTV: 85-90% of the prescription dose
  Dose to 97% of PTV: 90-97% of the prescription dose
  Volume of PTV > 115% of prescription dose: 1-5%
  Volume of PTV > 110% of prescription dose: 10-20%
- Unacceptable (Major) Deviations:

  Prescription Dose (Intact): < 40 Gy or >50.4 Gy
  Prescription Dose (Postoperative): < 45.0 Gy or >54.0 Gy
  Prescription Dose (Brachytherapy): not per section 9.4
  99% isodose: encompasses < 85% of the PTV
  Dose to 99% of PTV: < 85% of the prescription dose
  Dose to 97% of PTV: < 90% of the prescription dose
  Volume of PTV > 115% of prescription dose: >5%

Volume of PTV > 110% of prescription dose: >20%

9.5.7 Normal Tissue Dose

- Per Protocol: according to guidelines as stated in sections 9.2.4.6, 9.4.2.4, 9.4.3.4, 9.4.4.4, and 9.4.5.4
- Unacceptable (Major) Deviations: not per sections 9.2.4.6, 9.4.2.4, 9.4.3.4, 9.4.4.4, and 9.4.5.4

9.5.8 Elapsed Days

- Per Protocol: ≤ 60 days
- Minor Deviation: > 60 days but ≤ 70 days
- Unacceptable (Major) Deviation: > 70 days

9.6 Radiation Adverse Events

Risks and side effects related to radiation therapy include:

Likely (more than 10%)

- Redness and skin irritation in the treatment area that may result in bleeding and/or infection, which may require hospitalization
- Loss of pubic hair in the treated area, usually temporary
- Tiredness
- Nausea and/or vomiting
- Sterility (inability to bear children) in fertile women
- Sterility (inability to produce children) in men

Less Likely (3-9%)

- Diarrhea
- Sores and bleeding from the bowel (these side effects may occur well after treatment and be serious enough to require surgery)
- Narrowing and dryness of the vagina (birth canal) and genital area with painful or difficult intercourse and possibly bleeding
- Development of extra tissue (fibrosis) in the anal canal, which may result in decreased function
- Long-term dryness of the skin
- Hip, pelvic, or sacral fracture
- Build up of fluid in ankles, feet, and/or legs

Rare, but serious (less than 2%)

- Narrowing or blockage of the bowel (these side effects may occur well after treatment and be serious enough to require surgery)
- Blockage of the urinary tubes
- Development of an abnormal path or connection between organs (fistulae)
- Skin damage (tissue death), which may result in surgery
- Narrowing of or persistent bleeding in the vagina (birth canal), which may result in surgery

9.7 Functional Bone Marrow Sparing Substudy

All subjects will undergo PET/CT simulation with a slice thickness of 3 mm and large field-of-view pelvic protocol. Subjects imaged with FDG-PET will undergo intravenous administration of 200-400 MBq of ^{18}F-FDG 60 minutes prior to simulation. Subjects imaged with FLT-PET will undergo intravenous administration of 4.5 MBq/kg of ^{18}F-FLT 60 minutes prior to simulation.

10.0 DRUG THERAPY

10.1 Cisplatin

10.1.1 Source and Formulation: Cisplatin is commercially available from Bristol-Myers Oncology and from Pfizer as a dry powder supplied in 10 mg and 50 mg vials, and in aqueous solution in 50 mg and 100 mg vials with 100 mg mannitol and 90 mg sodium chloride; 10 mg/vial. The 10 mg and 50 mg vials should be reconstituted with 10 mL or 50 mL sterile water for injection USP, respectively. Each mL of the resulting solution will contain 1 mg of cisplatin. Reconstitution of powder results in a clear colorless solution when completed as recommended. NOTE: Aluminum reacts with cisplatin causing precipitation formation and loss of potency; therefore, needles or intravenous sets containing aluminum parts that may come in contact with the drug must not be used for the preparation or administration of cisplatin.

10.1.2 Administration: Patients will be prehydrated per institutional guidelines. Cisplatin will be dissolved at a concentration of 1 mL of sterile water/mg of drug, and the solution will be administered intravenously over 45-60 minutes. Supportive treatment will be given according to institutional policy.

10.1.3 Storage and Stability: Store at 15º to 20ºC. Unopened vials of dry powder are stable for the lot life indicated on the package when stored at room temperature. The aqueous solution should be stored at room temperature and protected from light. The reconstituted solution is stable for 20 hours at room temperature. Do not refrigerate. The cisplatin remaining in the amber vial following initial entry is stable for 28 days protected from light or for 7 days under fluorescent room light. NOTE: Once reconstituted, the solution should be kept at room temperature. If the reconstituted solution is refrigerated, a precipitate will form.

10.1.4 Adverse Events: Incidence rates of adverse events associated with cisplatin are provided in the product package insert. The following events are expected with the administration of cisplatin:

- Nephrotoxicity: Dose-related and cumulative renal insufficiency is the major dose-limiting adverse events of cisplatin. Renal adverse events have been noted in 28-36% of patients treated with a single dose of 50 mg/m2. It is first noted in the second week after a dose and is manifested as elevated BUN, creatinine, and serum uric acid, or as a decrease in creatinine clearance. Because renal adverse events become more prolonged and severe with repeated courses of cisplatin, renal function must return to normal before another dose can be given.
- Ototoxicity: Ototoxicity has been observed in up to 31% of patients treated with a single dose of cisplatin 50 mg/m2. It is manifested by tinnitus and/or hearing loss in the high frequency range. Deafness has been reported rarely.
- Hematologic Toxicity: Myelosuppression occurs in 25-30% of patients treated with cisplatin. Nadirs in circulating platelets and leukocytes occur

between Days 18 and 23 with most patients recovering by Day 39. Thrombocytopenia, anemia, neutropenia, and fever are also possible adverse events.

- Gastrointestinal Toxicity: Marked nausea and vomiting occur in almost all patients treated with cisplatin. Diarrhea and anorexia have also been reported.
- Neurotoxicity: Neurotoxicity usually characterized by peripheral neuropathies, has been reported. Neuropathy usually occurs after prolonged therapy (4 to 7 months); however, symptoms have been reported after a single dose. Muscle cramps, loss of taste, seizures, autonomic neuropathy, dorsal column myelopathy, and Lhermitte's sign have also been reported.
- Ocular Toxicity: Optic neuritis, papilledema, and cerebral blindness have been reported infrequently in patients receiving standard recommended doses of cisplatin. Blurred vision and altered color perception have been reported after the use of regimens with higher doses of cisplatin or greater dose frequency than those recommended in the package insert.
- Anaphylactic-like Reactions: Anaphylactic-like reactions have occasionally been reported in patients previously exposed to cisplatin. Symptoms include facial edema, wheezing, tachycardia, and hypotension.
- Hepatotoxicity: Transient elevations in liver enzymes, especially SGOT (AST), and bilirubin, have been reported.
- Other Toxicities: Other infrequent toxicities that have been reported include cardiac abnormalities, hiccups, elevated serum amylase, rash, alopecia, malaise, and asthenia. Rare cases of local soft tissue adverse events have occurred.

10.1.5 Mechanism of Action: Primarily causes inhibition of DNA synthesis and, to a lesser degree, inhibition of RNA and protein; it has not been shown to be cell cycle specific.

10.1.6 Pharmaceutical data: Cisplatin (cis-diamminedichloroplatinum II) has the empiric formula N2CL2 PtH6. It is a planar inorganic compound with a molecular weight of 300; soluble in water at a concentration of 1 mg/mL. The (II) nomenclature denotes the active valence state of the platinum. The interatomic distance of the chlorides is 3.3A, which is different from the 5-7A interatomic distance of the classic alkylating agents. Only the cis-isomer is therapeutically active.

10.1.7 Supply: This drug is commercially available.

10.1.8 Duration of administration: Cisplatin 40 mg/m^2 (80 mg max) will be administered once weekly during external beam RT (5 cycles only). It is recommended that cisplatin be started on day 1 of external beam RT but it is acceptable to give cisplatin on days 1, 2, or 3 of external beam RT. Dose modifications and indications for holding cisplatin are provided below.

10.2 Dose Modification

10.2.1 Chemotherapy will be held for:

- ANC < 500 /mm^3.

- Platelets < 50,000/mm^3.
- Febrile neutropenia or bleeding.
- Persistent (>24 hours) grade 3 or 4 nausea and vomiting.
- Renal Failure (creatinine > 2.0 mg% or creatinine clearance < 50 ml/min).

10.2.2 Cisplatin will be resumed at a dose of 30 mg/m^2 (60 mg max) after the resolution of:

- Persistent (>24 hours) grade 3 or 4 nausea and vomiting.
- Renal Failure (creatinine > 2.0 mg% or creatinine clearance < 50 ml/min).

Resolution of nausea means grade < 3. Resolution of renal failure means creatinine < 2.0 mg% or creatinine clearance > 50 ml/min.

10.2.3 Cisplatin will be held for neurotoxicity grade 2 or higher and may resume only if resolving to grade 1 or lower. Cisplatin will be held for ototoxicity grade 2 or higher and will resume only if ototoxicity resolves grade 1 or lower. External radiation should continue while cisplatin is being withheld.

10.2.4 For persistent renal insufficiency, neurotoxicity, or ototoxicity, it is acceptable to replace cisplatin with carboplatin per institutional policy. Otherwise, cisplatin should be discontinued.

11.0 SURGERY

Patients who have undergone radical hysterectomy or who have had cervical carcinoma diagnosed following inadvertent simple hysterectomy are eligible for the study. Planned adjuvant extrafascial hysterectomy is not allowed.

12.0 OTHER THERAPY

12.1 Permitted Supportive Therapy/Procedures:

12.1.1 Antiemetics
12.1.2 Antidiarrheals
12.1.3 Analgesics
12.1.4 Nutritional and fluid supplementation
12.1.5 Myeloid growth factors
12.1.5 Packed red blood cell transfusions

12.2 Non-permitted Supportive Therapy

12.2.1 Erythropoieitic growth factors (e.g. epoetin alfa, epoetin beta, darbepoetin alfa, etc.

13.0 PATHOLOGY AND TISSUE / SPECIMEN SUBMISSION

13.1 Histologic Confirmation of Diagnosis

All patients must have pathologically confirmed cervical carcinoma by biopsy or surgical pathology.

13.1 Specimen Submission

No specimen submission is required for this protocol

14.0 PATIENT ASSESSMENTS, FOLLOW-UP, AND DATA COLLECTION PROCEDURES

14.1 General

This study will use a general website available for access to the most recent protocol, case report forms, translation documents, and instructional videos. All information can be accessed at: http://radonc.ucsd.edu/research/irtoc/Pages/trials.aspx

Demographic, disease-related, treatment-related, toxicity, and outcomes data will be collected on standardized forms and submitted electronically to UC San Diego for central data processing via secure FTP server. Instructions for data transfer to UC San Diego can be found at http://radonc.ucsd.edu/research/irtoc/Pages/data-transfer.aspx. Data may be transmitted on scanned hard copies of each form (which are available on the IRTOC website) or may be conducted via electronic data submission (a protocol amendment with instructions for electronic data submission will be issued when this becomes available). Instructions on web registrations and electronic data submission will be posted at http://radonc.ucsd.edu/research/irtoc/Pages/trials.aspx. Copies of forms, including translated documents, may be downloaded from http://radonc.ucsd.edu/research/irtoc/Pages/forms.aspx

14.2 Time Schedule for Assessment, Follow-Up and Data Collection

Study Procedures	Pre-Treatment	With Each Fraction	Weekly During Treatment	Mid-Treatment (3rd week)	1 week, 2 weeks and 2 months post-treatment	1 month post-treatment[c]	Months post-treatment[d]
Informed Consent	X						
History and Physical	X					X	
Physical Exam with Vital Signs	X		X			X	6, 12, 18, 24, 30, 36
CBC with Differential[a]	X		X		X	X	6, 12, 18, 24, 30, 36
Comprehensive Metabolic Panel[ab]	X		X[e]			X	6, 12, 18, 24, 30, 36
Cervical / vaginal cytology	X						6, 12, 18, 24, 30, 36
Diagnostic chest x-ray, chest CT, or PET/CT	X						6, 12, 18, 24, 30, 36
Diagnostic pelvic CT, MRI, or PET/CT	X						6[f], 12, 18, 24, 30, 36
CT or PET/CT Simulation	X						
Pelvic MRI	Option-						

and/or PET/CT for radiation planning	al						
Baseline Data Collection (DHIF, DEF, TIF)	X						
Evaluation for Toxicity (STEF)	X		X		X	X	6, 12, 18, 24, 30, 36
Evaluation for Outcomes (OEF)						X	6, 12, 18, 24, 30, 36
Evaluation for Quality of Life (QOLA)	X					X	6, 12, 24, 36
kV CBCT (Substudy 2 only)		X					
MRI IDEAL-IQ (Substudy 3 only)	X			X	X[g]		6-9[h], 12[i]

[a] CBC with differential and Complete Metabolic Panel should be collected weekly during treatment (patient is considered on treatment until all radiation is complete)
[b] Electrolytes including Mg and Ca, Creatinine, Bilirubin, SGOT, and SGPT
[c] Visit should occur 1 month post completion of all radiation +/- 14 days
[d] Visit should occur at timepoints above +/- 14 days
[e] Weekly CMP during chemoradiation is strongly encouraged, but less frequent evaluation is acceptable in accordance with institutional policy.
[f] If feasible, post-treatment PET or PET/CT is strongly encouraged to assess for disease persistence
[g] Within 14 days following external beam radiation therapy
[h] 6-9 months following all therapy (external beam + brachytherapy)
[i] One-year study MRI should be obtained if funding permits

14.3 Demographic and Health Information Form
Baseline demographic and health data will be collected on a standard demographic and health information form (DHIF) and submitted for central data processing as described above.

14.4 Disease-Related Evaluation Form
Disease-related data will be collected on a standard disease evaluation form (DEF) and submitted for central data processing as described above.

14.5 Treatment-Related Information Form
Treatment-related data will be collected on a standard treatment information form (TIF) and submitted for central data processing as described above.

14.6 Symptom and Toxicity Evaluation Form

Toxicity data (i.e., anticipated adverse events) will be recorded on a standard symptom and toxicity evaluation form (STEF) and submitted for central data processing as described above.

14.7 Outcomes Evaluation Form

Outcomes data will be recorded on a standard outcomes evaluation form (OEF) and submitted for central data processing as described above

14.8 Quality of Life Assessment

Quality of life (QOL) data will be assessed using the EORTC QLQ-C30 and CX24 forms, which are combined together in a QOL assessment (QOLA) form (see Appendix IX). This form will be submitted for central data processing as described above. The expected peak acute GI and hematologic toxicity will occur within a month of completion of external beam radiation therapy. The justification for the QOL assessment schedule is based on clinical experience and the desire to gather representative data for both acute and late effects of chemoradiotherapy.

15.0 PATIENT SAFETY AND ETHICAL CONSIDERATIONS

15.1 Informed Consent

Written informed consent will be obtained prior to any study procedures (see section 8.2.2 and Appendix I).

15.2 Adverse Event Reporting

All expected adverse events will be documented on the Symptom and Toxicity Evaluation Form (STEF). Any unsuspected or confirmed unanticipated adverse event should be reported immediately (within 24 hours) to the study principal investigator (lmell@ucsd.edu) and Data Safety Monitoring Committee via the INTERTECC main email (irtoc@ucsd.edu). This study will utilize the Common Terminology Criteria for adverse events (CTCAE) of the National Cancer Institute for reporting of unanticipated serious adverse events (SAE). A copy of the CTCAE version 4.0 can be downloaded from the CTEP home page (http://ctep.cancer.gov/reporting/ctc.html). Unanticipated SAE information will be collected, documented, and reported comprehensively starting with entry into the study and continuing through 1 month post treatment. SAEs will be reported to the study principal investigator, the patient’s institution’s IRB, and the Data Safety and Monitoring Committee,. This reporting will include a description of the event, the subject’s study identification number, severity of the event, and its suspected cause. This form will include timing, severity and perceived causation of the events and followed until they either stabilize or resolve.

15.3 Steps to Determine If an Adverse Event is Reported in an Expedited Manner

Step 1: Identify the type of adverse event using the CTCAE v4. The CTCAE v4 provides descriptive terminology and a grading scale for each adverse event listed. A copy of the CTCAE v4 can be downloaded from the CTEP home page (http://ctep.cancer.gov/reporting/ctc.html). Additionally, if assistance is needed, the NCI has an Index to the CTCAE v4 that provides help for classifying and locating terms.

Step 2: Grade the adverse event using the NCI CTCAE v4.

Step 3: Determine whether the adverse event is related to the protocol therapy
Attribution categories are as follows:

Unrelated, Unlikely Related, Possibly Related, Probably Related, and Definitely Related.
Note: This includes all events that occur within 30 days of the last day of protocol treatment. Any event that occurs more than 30 days after the last day of treatment and is attributed (possibly, probably, or definitely) to the treatment must also be reported according to the instructions above.

Step 4: Determine the prior experience of the adverse event. Expected events are those that have been previously identified as resulting from administration of the treatment. An adverse event is considered unexpected, for expedited reporting purposes only, when either the type of event or the severity of the event is not listed in:

- the current known adverse events listed in the STEF of this protocol ;
- the investigator's brochure or the drug package insert

Step 5: Review Section 15.2 to determine if:
- there are any protocol-specific requirements for expedited reporting of specific adverse events that require special monitoring; and/or
- there are any protocol-specific exceptions to the reporting requirements.

15.4 Reporting Requirements for Serious Adverse Events

The investigator will report all unexpected SAEs that are determined to be possibly, probably or definitely related to the research within 10 working days of learning of the event to both the principal investigator (lmell@ucsd.edu) and the Data and Safety Monitoring Committee via the INTERTECC main email (irtoc@ucsd.edu).

Toxicities from Experimental Treatment
- Death on Study - regardless of cause: Written Report
- Grade 4 anaphylaxis: Written report
- All other expected toxicities, grades 1-4: Report in Continuing Review.

15.5 Criteria for Removal from Protocol Therapy

a) Unacceptable toxicity requiring removal from study (see section 15.2).
b) Non-compliance that in the opinion of the investigator does not allow for ongoing participation.
c) Physician determines it is in the subject's best interest.
d) Patient refusal of protocol therapy

15.6 Criteria for Removal from Study

a) Loss to follow-up.
b) Withdrawal of consent for any further data submission.

15.7 Data and Safety Monitoring Plan

The Data and Safety Monitoring Committee at UC San Diego will assume the responsibility for the data and safety monitoring. All adverse events will be reported to the IRB in accordance with regulations. Confidentiality of data will be maintained through subject de-identified numbers, the use of locked cabinets to store paper records, and a secure database which is maintained at the Moores UC San Diego Cancer Center.

Study data will be collected and managed using REDCap electronic data capture tools hosted at the Clinical and Translational Research Institute at UC San Diego. REDCap (Research Electronic Data Capture) is a secure, web-based application designed exclusively to support data capture for research studies. REDCap provides: 1) an intuitive interface for data entry (with data validation); 2) audit trails for tracking data manipulation and export procedures; 3) automated export procedures for seamless data downloads to common statistical packages (SPSS, SAS, Stata, R); 4) procedures for importing data from external sources; 5) tools for on-the-fly generation of plots, descriptive statistics, and reports for study monitoring and quality assurance; and 6) advanced features, such as branching logic and calculated fields [83]. Creation of the forms via REDCap's web interface will be conducted by Dr. Mell and Dr. Boxwala in conjunction with the Clinical and Translational Research Institute (CTRI) Biostatics team at UC San Diego.

15.8 Confidentiality Procedures

All acquired data will be stored at UC San Diego on a secure password-protected server with access limited to the principal investigator, co-investigators, clinical trial coordinator, and authorized personnel (e.g., post-doctoral researchers, etc.). The database will be de-identified by removing subjects' names and only maintaining the subjects' medical record numbers as a unique identifier. Similarly, data acquired locally at each participating institution will be stored on a secure password-protected server with access limited to the site principal investigator, co-investigators, and authorized personnel. Deliberations and recommendations of the Data and Safety Monitoring Committee are strictly confidential. Each member of the Data and Safety Monitoring Committee must sign a statement of confidentiality.

15.9 Oversight of Other Centers and Dissemination of Information

Whenever there is an adverse event reported or a protocol amendment or change in the consent form or interim results or other information that may impact the risks to subjects or others, the study PI and/or coordinator at UC San Diego will notify all the institution's PI's and study coordinators via email.

16.0 STATISTICAL CONSIDERATIONS

16.1 Definition of Primary Endpoint

A "primary event" is defined as either: (1) acute grade ≥ 3 neutropenia or (2) "clinically significant" grade ≥ 2 small bowel toxicity (diarrhea). Grading is defined on the STEF (see Appendix VII). "Clinically significant" small bowel toxicity is defined as requiring intravenous fluids and/or combination opiate/anticholinergic antidiarrheal medication (e.g. diphenoxylate/atropine or equivalent). Documentation of the use of opiate/anticholinergic medication or intravenous fluids to treat diarrhea is required to score a primary small bowel toxicity event. "Acute" is defined as occurring on or after day 1 of treatment and within 1 month of conclusion of external beam radiotherapy.

16.2 Sample Size and Study Duration

16.2.1 The observed rate of acute grade ≥ 3 hematologic toxicity or acute grade ≥ 3 gastrointestinal toxicity aggregated from 7 large studies of concurrent cisplatin-based chemotherapy and conventional RT (standard treatment) is approximately 30% [4-8,84-85]. The observed rate of acute grade ≥ 3 hematologic toxicity or acute grade ≥ 2 gastrointestinal toxicity from the same set of studies is approximately 50%. We expect the probability of a primary event with standard therapy to be approximately 40%. Based

on studies in the literature [21-23,27,86-87] we predict that IMRT can reduce the probability of a primary event by approximately one half.

For the phase II study we will test the null hypothesis H_0: p=0.40 versus the one-tailed alternative hypothesis H_A: $p < 0.40$ (α=0.05; β=0.10). The sample size of 91 for phase II is determined based on an expected maximum probability of a primary event of 25% with the experimental treatment and allows for loss of up to 10% of enrolled patients for evaluation of the primary endpoint, using a one-sample binomial test.

For the phase III study we will test the null hypothesis that the probability of a primary event in the control arm (p0) and experimental arm (p1) are the same, i.e. H0: p0 = p1, versus the two-tailed alternative hypothesis HA: p0 ≠ p1 (□=0.05; β=0.20). The planned sample size for phase III is 334, using a two-sample binomial test, but the sample size will be adjusted based on the observed toxicity in phase II and will be powered for an absolute risk reduction of 15% in the rate of primary endpoint events between the two arms. The sample size adjustment and phase III analysis plan will also consider site-to-site variability. A protocol amendment will be written to adjust the analysis plan, randomization procedures, stopping rules, and sample size for the phase III component of the trial. Feasibility of the phase III trial will be assessed based on the revised sample size and available funding.

16.2.2 The study duration will be 5 years (approximately 1 year for the phase II component and 4 years for the phase III component). The accrual period will be 4-5 years and the expected accrual rate is 8-10 patients per month. Subjects will be followed for long-term outcomes and toxicity for up to three years depending on available funding.

16.2.3 There is an accrual cap for each institution (see section 8.2.5).

16.2.4 For the phase III component, patients will be randomized at the time of registration. Randomization will be done at the CTRI with stratification on post-operative versus intact cervix status, using randomized blocks of size 4.

16.2.5 Accrual goals are 50 patients for the CBCT sub-study and 50 patients for the functional BM-sparing IMRT sub-study. A sample size of 50 results in approximately 80% power (one-sided α=.05) to detect a 50% relative reduction in the rate of grade ≥ 3 HT with functional BM-sparing IMRT. Based on our preliminary studies,[73-75] a sample size of 50 will provide an estimate of the optimal planning margin size with a 95% confidence interval within ± 2 mm.

16.2.6 Based on prior experience and published studies, we expect minimum rates of compliance with the QOL portion of the study to be 70% for this population. Estimated samples sizes for QOL assessments by study phase, assuming 70%, 80% and 90% compliance are:

Study Phase	70%	80%	90%
Phase II	64	73	82
Phase III	234	267	301
Total (phase II and III)	298	340	383

<u>16.3 Plan for Data Analysis</u>

16.3.1 The primary aim of the study is to test the hypothesis that IMRT reduces the probability of a primary hematologic or small bowel toxicity event compared to standard techniques in cervical cancer patients undergoing concurrent cisplatin and pelvic radiotherapy. This hypothesis will be tested using a one-sided Wilson score test with a with a null hypothesis H_0: p=0.40 and alternative hypothesis H_A: $p < 0.40$ (α=0.05).

16.3.2 Feasibility of IMRT at a given institution will be defined as the ability of the participating institution to treat at least 80% of its enrolled patients per protocol. Per protocol is defined as both (1) completing the entire treatment course within 60 days and (2) meeting the dosimetric and targeting criteria defined in section 9.5. If no institution is able to meet the feasibility criteria then this protocol will be deemed infeasible. If each of the 25 participating sites has a 90% probability of meeting the feasibility definition, the probability that the protocol will be deemed feasible is 1-(90%)^25 = 93%. Acute and late toxicity and adverse events, outcomes, and quality of life will be assessed using instruments described in section 14.0. Chemotherapy delivery will be evaluated in terms of numbers of cycles and total cumulative dose delivered. Feasibility of IMRT at each institution will be assessed at the end of phase II. If feasibility is not shown, then eligibility to enroll on phase III will be suspended.

16.3.3 Time to recurrence will be defined as the time from study enrollment to the time of recurrence of disease, determined by biopsy or imaging. If biopsy is not obtained to confirm diagnosis of recurrence, treatment failure will be determined to have occurred only if the patient undergoes a change in management on the basis of the imaging results (e.g., salvage or palliative therapy or hospice referral). Local recurrence will be defined as persistence or recurrence of tumor at the cervix or vaginal cuff. Regional recurrence will be defined as recurrence of tumor elsewhere in the pelvis or para-aortic nodal region (if treated). Distant recurrence will be defined as recurrence of tumor outside of the treatment field, including the para-aortic nodal region. Time to locoregional recurrence will be defined as the shortest time to local or regional recurrence, whichever occurs first. Time to recurrence will be defined as the shortest time to locoregional or distant recurrence, whichever occurs first. The cumulative probability of recurrence will be estimated using cumulative incidence functions, considering other first events (such as deaths) as competing events.

16.3.4 Disease-free survival will be defined as freedom from recurrence or death. Disease-specific survival will be defined as freedom from death due to cervical cancer. Overall survival will be defined as freedom from death due to any cause. These outcomes will be estimated using Kaplan-Meier survival curves with 95% confidence bands. Final outcomes will be assessed at the conclusion of the study, three years after enrollment of the last patient.

16.3.5 Planning margin estimates from CBCT sub-study
For each patient, the CTV will be contoured on each of the patient's CBCTs as described previously (section 9.2.2). The CBCTs will be rigidly registered to the planning CT and the CTVs for each CBCT will be cast onto the planning CT using methods described previously [73]. The minimum uniform margin around the planning CTV required to entirely encompass the CTV on each CBCT will be calculated. The data will be analyzed using linear mixed-effects models to estimate the margin required to encompass the CTV with 95% probability in the population, as described previously [73-75]. The mixed-effects models will include patient-specific random intercepts to account for intra-patient correlation of the repeated measures.

16.3.6 Analysis of the phase III data will be according to intention to treat. Phase II data will not be combined with the phase III data for analysis, since the phase II study is non-randomized. If significant site-to-site variability is observed, we will plan to model this in phase III using frailty or mixed-effects models.

16.3.7 QOL data will be analyzed separately by phase of study. The focus of phase II is primarily feasibility, i.e. compliance and scoring consistency across languages. The analysis of the phase III longitudinal QOL data will use linear random effects modeling, adjusting for important covariates such as the baseline QOL in addition to the treatment assignment. Nested random effects can also be used to model site-to-site variation. Non-informative missingness in the data is naturally accommodated by the random effects models. The QOL data at 1 month post treatment will be correlated with peak toxicities which occur during that month.

16.4 Planned Subgroup Analyses

Primary and secondary endpoints will be analyzed according to several factors, as listed in the schema (section 1.0). Differences in proportions will be tested using the chi square or t test. Differences in time to events will be analyzed using the log rank test or Gray's test and/or inclusion as covariates in multi-variable Cox proportional hazards models.

16.5 Missing data

Differences in baseline characteristics between those lost to follow-up and those completing the protocol will be assessed using chi-square or t-test. A worst-case sensitivity analysis will be conducted to assess whether findings are robust to the missing data.

16.6 Site effects

We will use logistic and proportional hazards mixed-effects regression models to test for and, as necessary, model site effects on safety and efficacy outcomes [88-89].

16.7 Sub-study analyses

Substudy 1 will test the primary hypothesis that patients with increased radiation dose to "critical" bone marrow subregions, as defined by Liang et al. [47] using high-dimensional data analysis, have a higher probability of acute hematologic toxicity. The hypothesis will be tested using a generalized linear model. Substudy 2 will test the primary hypothesis that 95% of target volumes will be encompassed within an optimal planning surface defined by a high-dimensional shape model developed by Khan et al. [75]. Substudy 3 will test the primary hypothesis that patients treated with functional BM-sparing IMRT have lower rates of hematologic toxicity than patients treated with standard IMRT and conventional RT techniques using a Mann-Whitney U test.

All subjects' data will be used in substudy 1. Substudy 2 will be limited to data collected from sites submitting daily CBCT data. Substudy 3 will be limited to data collected from centers able to administer functional BM-sparing IMRT. Centers will declare in advance whether they intend to enroll patients on the daily CBCT substudy and/or the functional BM-sparing IMRT substudy.

17.0 REFERENCES

1. Jemal A, Bray F, Center MM, Ferlay J, Ward E, Forman D. Global cancer statistics. *CA Cancer J Clin* 2011;61:69-90.

2. WHO GLOBOCAN 2002 database: http://www-dep.iarc.fr/, accessed May 10, 2010.
3. Keys HM, Bundy BN, Stehman FB, et al. Cisplatin, radiation, and adjuvant hysterectomy compared with radiation and adjuvant hysterectomy for bulky stage IB cervical carcinoma. *N Engl J Med* 1999;340:1154-61.
4. Morris M, Eifel PJ, Lu J, et al. Pelvic radiation with concurrent chemotherapy compared with pelvic and para-aortic radiation for high-risk cervical cancer. *N Engl J Med* 1999;340:1137-43.
5. Eifel PJ, Winter K, Morris M, et al. Pelvic irradiation with concurrent chemotherapy versus pelvic and para-aortic irradiation for high-risk cervical cancer: an update of radiation therapy oncology group trial (RTOG) 90-01. *J Clin Oncol* 2004;22:872-80.
6. Peters WA 3rd, Liu PY, Barrett RJ 2nd, et al. Concurrent chemotherapy and pelvic radiation therapy compared with pelvic radiation therapy alone as adjuvant therapy after radical surgery in high-risk early-stage cancer of the cervix. *J Clin Oncol* 2000;18:1606-13.
7. Whitney CW, Sause W, Bundy BN, et al. Randomized comparison of fluorouracil plus cisplatin versus hydroxyurea as an adjunct to radiation therapy in stage IIB-IVA carcinoma of the cervix with negative para-aortic lymph nodes: a Gynecologic Oncology Group and Southwest Oncology Group study. *J Clin Oncol* 1999;17:1339-48.
8. Rose PG, Bundy BN, Watkins EB, et al. Concurrent cisplatin-based radiotherapy and chemotherapy for locally advanced cervical cancer. *N Engl J Med* 1999;340:1144-53.
9. Green JA, Kirwan JM, Tierney JF, et al. Survival and recurrence after concomitant chemotherapy and radiotherapy for cancer of the uterine cervix: a systematic review and meta-analysis. *Lancet* 2001;358:781-62.
10. Green J, Kirwan J, Tierney J, et al. Concomitant chemotherapy and radiation therapy for cancer of the uterine cervix. *Cochrane Database Syst Rev* 2005;(3):CD002225.
11. Long HJ, Bundy BN, Grendys EC Jr, et al. Randomized phase III trial of cisplatin with or without topotecan in carcinoma of the uterine cervix: a Gynecologic Oncology Group Study. *J Clin Oncol* 2005;23:4626-33.
12. Gatcliffe TA, Tewari KS, Shah A, et al. A feasibility study of topotecan with standard-dose cisplatin and concurrent primary radiation therapy in locally advanced cervical cancer. *Gynecol Oncol* 2009;112:85-9.
13. Dueñas-Gonzalez A, Cetina-Perez L, Lopez-Graniel C, et al. Pathologic response and toxicity assessment of chemoradiotherapy with cisplatin versus cisplatin plus gemcitabine in cervical cancer. *Int J Radiat Oncol Biol Phys* 2005;61:817-823.
14. Dueñas-González A, Zarbá JJ, Patel F, et al. Phase III, open-label, randomized ctudy comparing concurrent gemcitabine plus cisplatin and radiation followed by adjuvant gemcitabine and cisplatin versus concurrent cisplatin and radiation in patients with stage IIB to IVA carcinoma of the cervix. *J Clin Oncol* 2011;29:1678-85.
15. Roeske JC, Lujan A, Rotmensch J, Waggoner SE, Yamada D, Mundt AJ. Intensity-modulated whole pelvic radiation therapy in patients with gynecologic malignancies. *Int J Radiat Oncol Biol Phys*. 2000;48:1613-21.
16. Lujan AE, Mundt AJ, Yamada SD, et al. Intensity-modulated radiotherapy as a means of reducing dose to bone marrow in gynecologic patients receiving whole pelvic radiotherapy. *Int J Radiat Oncol Biol Phys* 2003;57:516–521.
17. Mell LK, Tiryaki H, Ahn KH, Mundt AJ, Roeske JC, Aydogan B. Dosimetric comparison of bone marrow-sparing intensity-modulated radiotherapy versus conventional techniques for treatment of cervical cancer. *Int J Radiat Oncol Biol Phys* 2008;71:1504-10.
18. Mundt AJ, Lujan AE, Rotmensch J, et al. Intensity-modulated whole pelvic radiotherapy in women with gynecologic malignancies. *Int J Radiat Oncol Biol Phys*. 2002;52:1330-7.

19. Mundt AJ, Mell LK, Roeske JC. Preliminary analysis of chronic gastrointestinal toxicity in gynecology patients treated with intensity-modulated whole pelvic radiation therapy. *Int J Radiat Oncol Biol Phys* 2003;56:1354-60.
20. Brixey CJ, Roeske JC, Lujan AE, et al. Impact of intensity modulated radiotherapy on acute hematologic toxicity in women with gynecologic malignancies. *Int J Radiat Oncol Biol Phys* 2002;54:1388–1396.
21. Roeske JC, Bonta D, Mell LK, Lujan AE, Mundt AJ. A dosimetric analysis of acute gastrointestinal toxicity in women receiving intensity-modulated whole-pelvic radiation therapy. *Radiother Oncol* 2003;69:201-7.
22. Mell LK, Kochanski JD, Roeske JC, et al. Dosimetric predictors of acute hematologic toxicity in cervical cancer patients treated with concurrent cisplatin and intensity-modulated pelvic radiotherapy. *Int J Radiat Oncol Biol Phys* 2006;66:1356–1365.
23. Rose BS, Aydogan B, Yeginer M, et al. Normal tissue complication probability modeling of acute hematologic toxicity in cervical cancer patients treated with chemoradiotherapy. *Int J Radiat Oncol Biol Phys* 2010; 2011;79:800-7.
24. Chen MF, Tseng CJ, Tseng CC, Yu CY, Wu CT, Chen WC. Adjuvant concurrent chemoradiotherapy with intensity-modulated pelvic radiotherapy after surgery for high-risk, early stage cervical cancer patients. *Cancer J* 2008;14:200-6.
25. Kidd EA, Siegel BA, Dehdashti F, et al. Clinical outcomes of definitive intensity-modulated radiation therapy with fluorodeoxyglucose-positron emission tomography simulation in patients with locally advanced cervical cancer. *Int J Radiat Oncol Biol Phys* 2010; 2010;77:1085-91.
26. Hasselle MD, Rose BS, Kochanski JD, et al. Clinical outcomes of intensity modulated pelvic radiation therapy for carcinoma of the cervix. *Int J Radiat Oncol Biol Phys* 2010; in press.
27. Jhingran A, Winter K, Portelance L, et al. A phase II study of intensity modulated radiation therapy (IMRT) to the pelvis for post-operative patients with endometrial carcinoma (RTOG 0418) (abstr.) *Int J Radiat Oncol Biol Phys.* 2008;72:S16-7.
28. http://clinicaltrials.gov/ct2/show/NCT00193804, accessed September 4, 2009
29. Greimel E, Thiel I, Peintinger F, Cegnar I, Pongratz E. Prospective assessment of quality of life of female cancer patients. Gynecol Oncol. 2002;85:140-7.
30. Nout RA, van de Poll-Franse LV, Lybeert ML, et al. Long-term outcome and quality of life of patients with endometrial carcinoma treated with or without pelvic radiotherapy in the Post Operative Radiation Therapy in Endometrial Carcinoma 1 (PORTEC-1) Trial. *J Clin Oncol* 2011;29:1692-700.
31. Nout RA, Putter H, Jürgenliemk-Schulz IM, et al. Quality of life after pelvic radiotherapy or vaginal brachytherapy for endometrial cancer: first results of the randomized PORTEC-2 trial. *J Clin Oncol* 2009;27:3547-56.
32. Aaronson NK, Ahmedzai S, Bergman B, et al., for the EORTC Study Group on Quality of Life. The EORTC QLQ-C30. A quality of life instrument for use in international clinical trials in oncology. *J Natl Cancer Inst* 1993;85:365–375.
33. Scott NW, Fayers PM, Aaronson NK, Bottomley A, de Graeff A, Groenvold M, Koller M, Petersen MA, Sprangers MA; EORTC and the Quality of Life Cross-Cultural Meta-Analysis Group. The use of differential item functioning analyses to identify cultural differences in responses to the EORTC QLQ-C30. *Qual Life Res* 2007;16:115-29.
34. Greimel ER, Kuljanic Vlasic K, et al. The European Organization for Research and Treatment of Cancer (EORTC) Quality-of-Life questionnaire cervical cancer module: EORTC QLQ-CX24. *Cancer* 2006;107:1812-22.
35. Jayasekara H, Rajapaksa LC, Greimel ER. The EORTC QLQ-CX24 cervical cancer-specific quality of life questionnaire: psychometric properties in a South Asian sample of cervical cancer patients. *Psychooncology* 2008;17:1053-7.

36. Shin DW, Ahn E, Kim YM, et al. Cross-cultural application of the Korean version of the European Organization for Research and Treatment of Cancer quality of life questionnaire cervical cancer module. *Oncology* 2009;76:190-8.
37. Mauch P, Constine L, Greenberger J, et al. Hematopoietic stem cell compartment: Acute and late effects of radiation therapy and chemotherapy. *Int J Radiat Oncol Biol Phys* 1995;31:1319–1339.
38. Blomlie V, Rofstad EK, Skjonsberg A, et al. Female pelvic bone marrow: Serial MR imaging before, during, and after radiation therapy. Radiology 1995;194:537–543.
39. Fajardo LF, Berthrong M, Anderson RE. Hematopoietic tissue. In: Fajardo LF, Berthrong M, Anderson RE, editors. Radiation pathology. Oxford: Oxford University Press; 2001. p. 379–388.
40. Hall EJ, Giaccia AJ. Clinical response of normal tissues. In: Hall EJ, Giaccia AJ, editors. Radiobiology for the radiologist. 6th ed. Philadelphia: Lippincott Williams & Wilkins; 2006. p. 333–337; p. 461-2.
41. Rubin P, Landman S, Mayer E, et al. Bone marrow regeneration and extension after extended field irradiation in Hodgkin's disease. *Cancer* 1973;32:699–711.
42. Sacks EL, Goris ML, Glatstein E, et al. Bone marrow regeneration following large field radiation: Influence of volume, age, dose, and time. *Cancer* 1978;42:1057–1065.
43. Scarantino CW, Rubin P, Constine LS III. The paradoxes in patterns and mechanism of bone marrow regeneration after irradiation. 1. Different volumes and doses. *Radiother Oncol* 1984;2:215–225
44. Roeske JC, Mundt AJ. Incorporation of magnetic resonance imaging into intensity modulated whole-pelvic radiation treatment planning to reduce the volume of pelvic bone irradiated. Int Congress Series 2004;1268:307–312.
45. Roeske JC, Lujan A, Reba RC, et al.Incorporation of SPECT bone marrow imaging into intensity modulated whole-pelvic radiation therapy treatment planning for gynecologic malignancies. Radiother Oncol 2005;77:11-7.
46. Liang Y, Mell LK. Functional image-guided bone marrow-sparing intensity modulated radiation therapy for cervical cancer. In: Image-guided radiation therapy: a clinical perspective. Mundt AJ, Roeske JC (eds). Ontario: BC Decker. In press.
47. Liang Y, Messer K, Rose BS, et al. The impact of bone marrow radiation dose on acute hematologic toxicity in cervical cancer: principal component analysis on high dimensional data. *Int J Radiat Oncol Biol Phys* 2010; 78:912-9.
48. Vogler JB, Murphy WA. Bone marrow imaging. *Radiology*.1988; 168:679-93
49. Basu S, Houseni M, Bural G, et al.Magnetic resonance imaging based bone marrow segmentation for quantitative calculation of pure red marrow metabolism using 2-deoxy-2-[F-18]fluoro-D-glucose- positron emission tomography: a novel application with significant implications for combined structure-function approach. *Mol Imaging Biol* 2007. Piney A. The anatomy of the bone marrow with special reference to the distribution of the red marrow. *BMJ* 1922;28:792-795.
50. Hartsock RJ, Smith EB, Petty CS. Normal variations with aging of the amount of hematopoietic tissue in bone marrow from the anterior iliac crest. A study made from 177 cases of sudden death examined by necropsy. *Am J Clin Pathol* 1965;43:326-31.
51. Duda SH, Laniado M, Schick F, Strayle M, Claussen CD. Normal bone marrow in the sacrum of young adults: differences between the sexes seen on chemical-shift MR imaging. *AJR Am J Roentgenol* 1995;164:935-40.
52. Schick F, Lutz O. Assessment of the magnetic field distribution in yellow and red bone marrow by the MAGSUS technique. *Magn Reson Imaging* 1996;14:507-19.
53. Ellis RE. The distribution of active bone marrow in the adult. *Phys Med Biol* 1961;5:255–258.

54. Ricci C, Cova M, Kang YS, et al. Normal age-related patterns of cellular and fatty bone marrow distribution in the axial skeleton: MR imaging study. *Radiology* 1990;177:83Y88
55. Blebea JS, Houseni M, Torigian DA, et al. Structural and functional imaging of normal bone marrow and evaluation of its age-related changes. *Semin Nucl Med* 2007;37:185-94.
56. Reeder SB, Pineda AR, Wen Z, et al. Iterative decomposition of water and fat with echo asymmetry and least-squares estimation (IDEAL): application with fast spin-echo imaging. *Magn Reson Med* 2005;54:636-44.
57. Bydder M, Yokoo T, Hamilton G, et al. Relaxation effects in the quantification of fat using gradient echo imaging. *Magn Reson Imaging* 2008;26:347-59.
58. Yu H, McKenzie CA, Shimakawa A, et al. Multiecho reconstruction for simultaneous water-fat decomposition and T2* estimation. *J Magn Reson Imaging* 2007;26:1153-61.
59. Kijowski R, Woods MA, Lee KS, et al. Improved fat suppression using multipeak reconstruction for IDEAL chemical shift fat-water separation: application with fast spin echo imaging. *J Magn Reson Imaging* 2009;29:436-42.
60. Shields AF. Positron emission tomography measurement of tumor metabolism and growth: its expanding role in oncology. *Mol Imaging Biol* 2006;8:141-50.
61. Everitt S, Hicks RJ, Ball D, et al. Imaging cellular proliferation during chemo-radiotherapy: a pilot study of serial 18F-FLT positron emission tomography/computed tomography imaging for non-small-cell lung cancer. *Int J Radiat Oncol Biol Phys* 2009;75:1098-104.
62. Hayman JA, Callahan JW, Herschtal A, et al. Distribution of Proliferating Bone Marrow in Adult Cancer Patients Determined Using FLT-PET Imaging. *Int J Radiat Oncol Biol Phys* 2010. [Epub ahead of print]
63. Yue J, Chen L, Cabrera AR, et al.. Measuring tumor cell proliferation with 18F-FLT PET during radiotherapy of esophageal squamous cell carcinoma: a pilot clinical study. *J Nucl Med* 2010;51:528-34.
64. Menda Y, Ponto LL, Dornfeld KJ, et al. Investigation of the pharmacokinetics of 3'-deoxy-3'-[18F]fluorothymidine uptake in the bone marrow before and early after initiation of chemoradiation therapy in head and neck cancer. *Nucl Med Biol* 2010;37:433-8.
65. Koizumi M, Saga T, Inubushi M, et al. Uptake Decrease of Proliferative PET Tracer (18)FLT in Bone Marrow after Carbon Ion Therapy in Lung Cancer. *Mol Imaging Biol* 2010. In press.
66. van Waarde A, Jager PL, Ishiwata K, et al. Comparison of sigma-ligands and metabolic PET tracers for differentiating tumor from inflammation. *J Nucl Med* 2006;47:150-4.
67. van Waarde A, Cobben DC, Suurmeijer AJ, et al. Selectivity of 18F-FLT and 18F-FDG for differentiating tumor from inflammation in a rodent model. *J Nucl Med* 2004;45:695-700.
68. Rose BS, Liang Y, Lau SK, et al. Correlation between radiation dose to ^{18}F-FDG-PET defined active bone marrow sub-regions and acute hematologic toxicity in cervical cancer patients treated with chemoradiotherapy (abstr.) *Int J Radiat Oncol Biol Phys* 2011;
69. Ahamad A, D'Souza W, Salehpour M, et al. Intensity-modulated radiation therapy after hysterectomy: comparison with conventional treatment and sensitivity of the normal-tissue-sparing effect to margin size. *Int J Radiat Oncol Biol Phys* 2005;62:1117-24.
70. Buchali A, Koswig S, Dinges S, *et al*. Impact of the filling status of the bladder and rectum on their integral dose distribution and the movement of the uterus in the treatment planning of gynecological cancer. *Radiother Oncol* 1999;52:29-34.

71. van de Bunt L, Jürgenliemk-Schulz IM, de Kort GA, et al. Motion and deformation of the target volumes during IMRT for cervical cancer: what margins do we need? *Radiother Oncol* 2008;88:233-40.
72. Lim K, Kelly V, Stewart J, *et al*. Pelvic radiotherapy for cancer of the cervix: is what you plan actually what you deliver? *Int J Radiat Oncol Biol Phys* 2009;74:304-312.
73. Tyagi N, Lewis JH, Yashar CY, et al. Daily online cone beam computed tomography to assess inter-fraction motion in patients with intact cervical cancer. *Int J Radiat Oncol Biol Phys*. 2011;80:273-80.
74. Khan A, Jensen L, Sun S, et al. Optimal planning margins for intact cervical cancer (abstr.) *Int J Radiat Oncol Biol Phys* 2011
75. Khan A, Jensen L, Sun S, et al. Shape modeling for on-line adaptive radiotherapy in cervical cancer (abstr.) *Int J Radiat Oncol Biol Phys* 2011
76. Small W, Mell LK, Creutzberg C, et al. Consensus recommendations for intensity modulated radiation therapy planning for post-operative pelvic radiotherapy in endometrial cancer. Int J Radiat Oncol Biol Phys. 2008;71:428-34.
77. Lim K, Small W, Portelance L, et al. Consensus guidelines for delineation of clinical target volumes for intensity-modulated pelvic radiotherapy for the intact cervix (abstr.) *Int J Radiat Oncol Biol Phys.* 2009
78. http://www.rtog.org/atlases/gynatlas/main.html, accessed May 10, 2010.
79. Myerson RJ, Garofalo MC, et al. Elective clinical target volumes for conformal therapy in anorectal cancer: a radiation therapy oncology group consensus panel contouring atlas. *Int J Radiat Oncol Biol Phys*. 2009;74:824-30.
80. http://www.rtog.org/atlases/anoratlas/main.html, accessed May 10, 2010.
81. Haie-Meder C, Potter R, van Limbergen E, et al. Recommendations from Gynaecological (GYN) GEC-ESTRO Working Group() (I): concepts and terms in 3D image based 3D treatment planning in cervix cancer brachytherapy with emphasis on MRI assessment of GTV and CTV. *Radiother Oncol* 2005; 74(3):235-245.
82. Pötter R, Haie-Meder C, Limbergen EV, et al. Recommendations from gynaecological (GYN) GEC ESTRO working group (II): Concepts and terms in 3D image-based treatment planning in cervix cancer brachytherapy-3D dose volume parameters and aspects of 3D image-based anatomy, radiation physics, radiobiology. *Radiother Oncol* 2006; 78(1):67-77.
83. Harris PA, Taylor R, Thielke R, et al. Research electronic data capture (REDCap) - A metadata-driven methodology and workflow process for providing translational research informatics support, J Biomed Inform. 2009;42:377-81.
84. Pearcey R, Brundage M, Drouin P, et al. Phase III trial comparing radical radiotherapy with and without cisplatin chemotherapy in patients with advanced squamous cell cancer of the cervix. *J Clin Oncol* 2002;20:966-72.
85. Torres MA, Jhingran A, Thames HD Jr, et al. Comparison of treatment tolerance and outcomes in patients with cervical cancer treated with concurrent chemoradiotherapy in a prospective randomized trial or with standard treatment. *Int J Radiat Oncol Biol Phys* 2008;70:118-25.
86. Simpson DR, Song WY, Rose BS, et al. Normal tissue complication probability analysis of acute gastrointestinal toxicity in cervical cancer patients undergoing intensity modulated radiation therapy and concurrent cisplatin (abstr.) *Int J Radiat Oncol Biol* Phys 2010;78:S141-2.
87. Portelance L, Winter K, Jhingran A, et al. Post-operative pelvic intensity modulated radiation therapy (IMRT) with chemotherapy for patients with cervical carcinoma/RTOG 0418 phase II study (abstr.) *Int J Radiat Oncol Biol* 2009;75:S640-S641.
88. Stiratelli R, Laird N, Ware JH. Random-effects models for serial observations with binary response. *Biometrics* 1984; 40:961-971.

89. Vaida F, Xu R. Proportional hazards model with random effects. *Statistics in Medicine*, 2000;19:3309-24.

APPENDIX I: SAMPLE CONSENT FORM

University of California, San Diego
Consent to Act as a Research Subject

PHASE II/III CLINICAL TRIAL OF INTENSITY MODULATED RADIATION THERAPY WITH CONCURRENT CISPLATIN FOR STAGE I-IVA CERVICAL CARCINOMA

Protocol # __110808________________

Dr. Loren Mell and his colleagues are conducting a research study to find out more about a new radiation technique called "intensity modulated radiation therapy (IMRT)." You are being asked to take part because you have cervical cancer.

Your participation in this research study is voluntary. The purpose of this Informed Consent Form is to inform you about the nature of this research study so that you may make an informed decision as to whether you would like to participate. If you have any questions, please ask your study doctor or coordinator to explain any words or information that you do not understand.

PURPOSE

The purpose of this study is to find out whether patients with cervical cancer treated with IMRT have less side effects with equal cancer control compared to standard radiation techniques.

With standard radiation techniques, normal pelvic organs near your tumor receive radiation dose, which leads to side effects. IMRT is a new radiation technique that can reduce radiation dose to these organs and may reduce side effects.

Participation in this study is entirely voluntary. Approximately 425 patients will take part in this multi-center international study. Approximately 30 patients will be enrolled at UC San Diego. Your study doctor has no personal or financial interest in this research.

DURATION OF THE STUDY

The expected duration of this study is approximately 5 years.

IMAGING STUDIES

All imaging studies will be performed using standard techniques will be performed by authorized personal trained and licensed to perform the study or studies in question.

STANDARD TREATMENT AND PROCEDURES

Standard treatment for your disease consists of chemotherapy (cisplatin) and radiation therapy. The chemotherapy in this study (cisplatin) will be exactly the same as you will get if you do not participate in the study. The radiation therapy technique (IMRT) will be the only difference in your treatment. IMRT has been in use for cervical cancer for approximately 10 years but is not considered standard. As part of standard care, the following procedures may be required,

whether or not you choose to participate in this study:

Pretreatment Evaluations

After signing this consent form you will have to undergo the following tests and procedures to determine if you are a good candidate to participate further in the study. Depending on when you last had these tests and procedures performed, some of them may not need to be repeated.

- Medical and surgical history review (including medications that you are taking or have taken in the past.
- Complete physical examination, including height, weight & vital signs (blood pressure, heart rate, temperature, breathing rate).
- Blood samples will be taken for the following lab tests:
  - Hematology or CBC (Complete Blood Count), which includes: white blood cell count, red blood cell count, platelet count, hemoglobin (oxygen-carrying pigment in red blood cells), hematocrit (measures the amount of space red blood cells take up in the blood). This is to aid in diagnosing anemia (low red blood cell count which can result in fatigue), certain cancers of the blood, and to monitor blood loss and infection.
  - Blood chemistry (which measures the levels of a number of chemical substances that are released from various tissues in the body to evaluate the function of the liver and kidneys)
- Cervical/vaginal cytology
- Diagnostic Chest x-ray, chest CT, or PET/CT
- Diagnostic Pelvic CT scan or MRI
- CT or PET/CT simulation. This is when your study doctor will decide the best way to deliver the dose of radiation and limit the exposure of your vital organs.
- Pelvic MRI and/or PET/CT for radiation planning (optional)
- Baseline data collection (i.e. demographic data).
- Evaluation for toxicity (anticipated adverse events).
- Evaluation for Quality of Life.

Treatment Procedures

You will receive radiation therapy daily for 5 to 5½ weeks.
Once a week on study days 1, 8, 15, 22, and 29 you will receive intravenous infusions of cisplatin prior to your radiation therapy..

In addition, once a week you will have the following evaluations/tests done:
- Physical examination and vital signs
- Collection of blood samples for laboratory testing to check for normal organ function and to find out your disease status
- Review of any side effects

Post Treatment Procedures

At 1 week, 2 weeks and 2 months after your last study treatment you will return for the following procedures:
- Collection of blood samples for laboratory testing to check for normal organ function and to find out your disease status
- Review of any side effects

At 1 month after your last study treatment you will return for the following procedures:

- Medical and surgical history review (including medications that you are taking or have taken in the past.
- Complete physical examination, including height, weight & vital signs (blood pressure, heart rate, temperature, breathing rate).
- Collection of blood samples for laboratory testing to check for normal organ function and to find out your disease status
- Cervical/vaginal cytology
- Review of any side effects
- Evaluation for Outcomes
- Evaluation for Quality of Life.

At 4, 8, 12, 18, 24, 30, and 36 months after your last study treatment you will return for the following procedures:

- Physical examination and vital signs
- Collection of blood samples for laboratory testing to check for normal organ function and to find out your disease status
- Cervical/vaginal cytology
- Chest x-ray, chest CT, or PET/CT
- Pelvic CT scan or MRI to evaluate response to treatment and disease status
- Review of any side effects
- Evaluation for Outcomes
- Evaluation for Quality of Life.

Description of Procedures:

Tumor biopsy: A sample of your tumor is taken to confirm the type of cancer you have and what type of treatment is required.

CT scan: The CT scanner is a free-standing machine with a large hole in the center. You will be asked to lie on your back with your arms raised above your head on a narrow table that slides into the hole. Patients who have difficulty with enclosed spaces such as those found with some MRI scanners do not usually have a problem with this type of test. A dye may be injected into a peripheral vein to better evaluate certain diseases and organs. The radiologist will decide if this is necessary. Tell the technician or radiologist if you have any allergies or have had difficulty with prior CT scans. It is very important that you remain still throughout the exam and hold your breath when asked. This will allow for better images. The actual scan time is usually about two minutes, although the entire procedure usually takes much longer.

MRI: Magnetic Resonance Imaging (MRI) may be done to measure your tumor. This will involve lying quietly inside the center of a large, doughnut-shaped magnet for approximately 30-60 minutes.

Chest X-ray: This test is performed by an x-ray technician. You will be asked to stand in front of the machine and must hold your breath when the x-ray is taken.

Positron Emission Tomography (PET scan): You will be taken into a special injection room, where the radioactive substance is administered as an intravenous injection (although in some cases, it will be given through an existing intravenous line or inhaled as a gas). It will then take

approximately 30 to 90 minutes for the substance to travel through your body and accumulate in the tissue under study. During this time, you will be asked to rest quietly and avoid significant movement or talking. After that time, scanning begins. This may take 30 to 45 minutes.

Hemogram or CBC (Complete Blood Count), which includes: white blood cell count, red blood cell count, platelet count, hemoglobin (oxygen-carrying pigment in red blood cells), hematocrit (measures the amount of space red blood cells take up in the blood). This is to aid in diagnosing anemia (low red blood cell count which can result in fatigue), certain cancers of the blood, and to monitor blood loss and infection.

Blood chemistry: measures the levels of a number of chemical substances that are released from various tissues in the body to evaluate the function of the liver and kidneys

HIV test: in order to make sure that the study procedures are appropriate for you (HIV testing may require a separate consent form for you to sign. This form will be provided by the Study Doctor.)

RISKS OF STANDARD TREATMENT AND PROCEDURES

Standard treatment for your disease may involve risks and discomforts. You will be at risk for the side effects listed below, whether or not you choose to participate in this study. You should discuss these with your doctor. There may also be other side effects that we cannot predict. Medicines and other treatments can be given to make the side effects less serious and uncomfortable. Many side effects go away shortly, but in some cases, side effects may be serious, long-lasting, and may even cause death.

Risks and side effects related to <u>radiation therapy</u> include:

Likely (more than 10%)

- Redness and skin irritation in the treatment area that may result in bleeding and/or infection, which may require hospitalization
- Loss of pubic hair in the treated area, usually temporary
- Tiredness
- Nausea and/or vomiting
- Sterility (inability to bear children) in fertile women

Less Likely (3-9%)

- Diarrhea
- Sores and bleeding from the bowel (these side effects may occur well after treatment and be serious enough to require surgery)
- Narrowing and dryness of the vagina (birth canal) and genital area with painful or difficult intercourse and possibly bleeding
- Development of extra tissue (fibrosis) in the anal canal, which may result in decreased function
- Long-term dryness of the skin
- Hip or pelvic or sacral fracture
- Build up of fluid in ankles, feet, and/or legs

Rare, but serious (less than 2%)

- Narrowing or blockage of the bowel (these side effects may occur well after treatment and be serious enough to require surgery)
- Blockage of the urinary tubes
- Development of an abnormal path or connection between organs (fistulae)
- Skin damage (tissue death), which may result in surgery
- Narrowing of or persistent bleeding in the vagina (birth canal), which may result in surgery

Risks and side effects related to <u>chemotherapy (cisplatin)</u> include:

- Kidney toxicity
- Ringing in the ears and/or hearing loss
- Low blood counts
- Nausea
- Vomiting
- Diarrhea
- Low appetite
- Nerve injury
- Muscle cramps
- Loss of taste
- Seizures
- Blurry vision
- Altered color perception
- Allergic reactions (swelling, wheezing, increased heart rate, low blood pressure)
- Liver toxicity

- Other infrequent side effects including heart disease, hiccups, rash, hair loss, low energy, skin changes, or muscle cramps or pains.

Risk of Testing for HIV: As part of your treatment, you may be tested for HIV. These tests are necessary to make sure that the treatment is appropriate for you. Testing for HIV may result in a diagnosis of infection with this virus. You will be informed of the results of these tests; if you do not wish to know the results, then you may refuse to be tested if you choose. In the event that you are diagnosed with HIV, your doctor will give you the results in a face-to-face discussion (not by telephone or mail), counseling will be offered to you, and the results will be entered in your medical record and provided to the California State Board of Health. In the event that you are diagnosed with HIV, you may be referred to a doctor who specializes in these illnesses. The diagnosis of HIV may result in earlier treatment and/or prevention of many complications from the illnesses. Efforts will be made to keep your personal information confidential. Awareness of a diagnosis of these illnesses may have serious personal or social consequences. Some of these consequences include possible difficulty obtaining health insurance or employment, and difficulty traveling to some foreign countries.

Allergic Reactions: There is the chance that your treatment could cause an allergic reaction, which may include difficulty breathing, rash, flushing, weakness, dizziness, lightheadedness, and swelling.

Intravenous (IV) Injection Side Effects: If drugs or contrast leak from the vein it is given into, it may cause skin irritation at the needle site.

Risks of blood draws: There is a risk of discomfort or pain, bleeding, swelling and a small arm bruise and swelling when blood is drawn. Rarely, a clot or infection may occur at the site of the blood draw. Some people also become faint, dizzy, or light-headed during or immediately after the blood draw.

Reproductive Risks (this only applies if you are capable of becoming pregnant): You should not become pregnant while on this study because the drugs in this study can affect a fetus and cause serious birth defects. Women should not breastfeed a baby while on this study. The treatments used for your disease will make you unable to have children in the future. It is important you understand that if you are capable of child-bearing then you need to use birth control while on this study. If you capable of child-bearing, a pregnancy test will be done before the study begins in order to be as sure as possible that you are not pregnant. Your treatment requires that you use contraception methods (such as abstinence, diaphragm, condom, or intrauterine device) to prevent pregnancy for the duration of the study. Ask about counseling and more information about preventing pregnancy.

Risks from X-rays and/or Scans: As part of your treatment, you will have imaging scans. These tests are necessary to evaluate, plan the treatment for, and monitor your disease. As a result of participating in this study, you will be exposed to a significant amount of radiation from diagnostic tests (approximately 280 mSv). This amount is more than you would receive from a year of natural exposure in the San Diego area, which is approximately 1.6 mSv. Cumulative exposure from radiation may increase your chances of developing certain types of cancer in the future. The principal investigator for this research study had determined that all of the imaging prescribed for this study would typically be performed as part of standard medical care. Radiation exposure may be decreased if non-radiaiton alternatives are used, such as MRI. If you are especially concerned with radiation exposure, or you have had a lot of x-rays or imaging scans already, you should discuss this with the principal investigator of the study, Dr. Loren

Mell, or your regular doctor. Note that the exposure you will receive from diagnostic imaging is much less than the exposure you will receive from treatment, which is 45-50 Gy.

Risks of MRI Scans: As part of your treatment, Magnetic Resonance Imaging (MRI) may be done. The imager makes a loud, banging noise while it is taking pictures. You will be given a set of ear plugs to help with the noise. You may experience feelings of claustrophobia or anxiety. You may also experience some discomfort and tiredness from lying still in a confined space during the imaging. There are no known effects from exposure to magnetic fields (MRI). However, some patients undergoing this procedure become anxious. If this happens to you, you can stop the procedure at any time. If you have metal clips or plates in your body or a pacemaker, you should tell your doctor about it. MRI may not be appropriate under some of the following conditions: a cardiac pacemaker; metal fragments in eyes, skin, or body; heart valve replacement; brain clips; venous umbrella; being a sheet-metal worker or welder; aneurysm surgery; intercranial bypass; renal or aortic clips; prosthetic devices such as middle ear, eye, joint, or penile implants; joint replacements; hearing aid; neurostimulator; insulin pump; I.U.D.; being pregnant or trying to become pregnant; shunts/stents; metal mesh/coil implants; metal plate/pin/screws/wires, or any other metal implants; and permanent eyeliner and/or eyebrows.

Risks of IV Contrast: As part of your treatment, a CT scan may be done. There may be some reactions related to the contrast dye used in CT scans. Contrast dye is usually administered when you get a CT scan. Contrast dye may also be used in MRI scans. Some people may develop hives and itching or other allergic symptoms from this dye, swelling of the heart, cramps of the voicebox, breathing distress caused by narrowing of the airways in lungs, low blood pressure, with loss of consciousness, and in rare cases, severe loss of blood and fluids leading to shock and death, fainting, seizures, and irregular heartbeats. In addition, if you have low kidney function, this dye can temporarily or permanently decrease your kidney function.

BENEFITS OF PARTICIPATION IN THIS STUDY

If you agree to take part in this study, there may be a direct medical benefit to you. IMRT reduces dose to normal organs, which previous studies have indicated may reduce side effects compared to standard radiation therapy. However, the benefits of IMRT are unknown. Others may also benefit from the information learned from this research study.

RISKS OF PARTICIPATION IN THIS STUDY

Participation in this study may involve some added risks or discomforts, as explained below. There may also be other side effects that we cannot predict. Many side effects go away shortly, but in some cases, side effects may be serious, long-lasting, and may even cause death.

Side effects from IMRT, in addition to those of standard radiation therapy (listed above), may include:

- Second cancers (rare, less than 1%)

Risks from X-rays and/or Scans: If you participate in this study, you may receive additional imaging scans. These tests are necessary to ensure that your target is in the proper position each day of treatment. If you choose to participate in daily CT scan study, you will be exposed to a significant amount of radiation from these scans (approximately 250 mSv). This amount is more than you would receive from a year of natural exposure, which is approximately 1.6 mSv. This exposure may slightly increase your chances of developing cancer in the future. If you are

especially concerned with radiation exposure or you have had a lot of x-rays already, you should discuss this with your doctor. Note that exposure you will receive from these procedures is much less than the exposure you will receive from treatment, which is more than 45000 mSv.

ALTERNATIVES TO PARTICIPATION IN THIS STUDY

If you choose not to take part in or stop participating in this research study, there may be other treatments. Refusal to take part in this study will not cause penalty or loss of benefits to which you are otherwise entitled. You do not have to participate in this study to receive treatment for your cancer. Other possible treatments could include treatment with radiation, including IMRT, and other drugs or drug combinations, participation in other research studies, or supportive care only (no cancer treatment). Please talk to your doctor about these and other options.

COSTS/COMPENSATION

You or your insurance company/third party payor will be billed for all routine procedures and drugs associated with this study including the cost of treating injuries resulting from such routine procedures. Routine procedures and drugs are those that you would likely receive whether or not you are in this study. You will be responsible for any deductibles or co-payments that are associated with your insurance coverage. Examples of procedures and drugs that may be billed include the following: diagnostic tests, radiation therapy and chemotherapy (cisplatin). There will be no payment to you for participating in this study.

COMPENSATION FOR RESEARCH-RELATED INJURY

If you are injured as a direct result of participation in this research, the University of California will provide any medical care you need to treat those injuries. The University will not provide any other form of compensation to you if you are injured. You may call the Human Research Protections Program Office at (858) 455-5050 for more information about this, to inquire about your rights as a research subject or to report research-related problems.

VOLUNTARY PARTICIPATION

Participation in this study is entirely voluntary. If you choose not to participate or wish to withdraw your consent to participate in these study procedures at any time, it will in no way affect your regular treatments or medical care at this institution or loss of benefits to which you are entitled.

You will be informed of any new findings that might affect your willingness to continue participating in the study.

If health conditions occur which would make your participation in this study possibly dangerous, or if other conditions occur that would make participation in this study detrimental to you or your health, then your study doctor may discontinue this study procedure.

Your study doctor or sponsor may stop your participation in this study at any time without your consent if:

You have side effects that require removal from the study.
You do not comply with the procedures required for study participation
The doctor does not deem participation in the study to be in your best interest.
You refuse therapy according to the terms of the study protocol

DO YOU HAVE ANY QUESTIONS?

Dr. Mell and/or his co-investigator or coordinator has explained this study to you, and has answered your questions. You may contact Dr. Mell at (858) 822-6040. You may also call the hospital 24-hour paging system at (858) 657-7000 and ask for the radiation oncologist on-call. If you have other questions or research-related problems, you may call the Moores UCSD Cancer Center Clinical Trials Office at (858) 822-5354. If you have questions about your rights as a research participant, your participation in this study, and/or concerns about this study, you may call the UCSD Human Research Protections Program (a group of people who review the research study to protect your rights and welfare) at (858) 455-5050.

PROCEDURES TO WITHDRAW FROM THE STUDY

If you no longer want to participate you can let your doctor know or contact Dr. Mell at one of the numbers listed above and you will be withdrawn from the study.

CONFIDENTIALITY

The confidentiality of your research records will be maintained to the extent permitted by law. Your medical information will not be made publicly available unless disclosure is required by law or regulation.

Data obtained from this study may be published or given to regulatory authorities, including the Food and Drug Administration (FDA), Department of Health and Human Services (DHHS) agencies, the UCSD Institutional Review Board, the Moores UCSD Cancer Center Data and Safety Monitoring Board (DSMB) and other governmental agencies in the United States or other countries in which regulatory approval may be sought. Any potential risk of loss of confidentiality will be minimized.

OPTIONAL Procedures

You may be asked to participate in Image-Guided Bone Marrow-Sparing IMRT using FLT-PET scan and quantitative MRI during this research study. These images will be used to understand how targets move and how to better design radiation therapy plans.

There may be a direct benefit to you by consenting to this optional procedure, because the treatment may be given more accurately as a result of this imaging. There may also be a benefit from the medical knowledge gained to patients in the future. There are no additional costs to you for taking part in the additional sampling.

Your study doctor has no personal or financial interest in this research.

Having read and understood the above, and having had the chance to ask questions about these additional, optional procedures, please initial next to your response below:

______ I give permission to take part in FLT-PET optional procedure.

______ I do **not** give permission to take part in FLT-PET optional procedure.

______ I give permission to take part in quantitative MRI optional procedure.

______ I do **not** give permission to take part in quantitative MRI optional procedure.

______________________________ ________________
Signature of Participant Date

SIGNATURE AND CONSENT

Your participation in this study is voluntary, and you may refuse to participate or withdraw from the study at any time without prejudice or loss of benefits to which you are otherwise entitled. You will receive a signed copy of this consent document and a copy of "The Experimental Subject's Bill of Rights" to keep.

You agree to participate.

____
Printed Name of Participant

______________________________ ________________
Signature of Participant Date

____
Printed Name of Person Obtaining Consent

______________________________ ________________
Signature of Person Obtaining Consent Date

APPENDIX II: FIGO / AJCC CANCER STAGING SYSTEM, CERVIX (AJCC, 2010, 7th Edition)

Primary tumor (T)

TNM categories	FIGO stages	Definition
TX		Primary tumor cannot be assessed
T0		No evidence of primary tumor
Tis*		Carcinoma in situ (preinvasive carcinoma)
T1	I	Cervical carcinoma confined to uterus (extension to corpus should be disregarded)
T1a*	IA	Invasive carcinoma diagnosed only by microscopy. Stromal invasion with a maximum depth of 5.0 mm measured from the base of the epithelium and a horizontal spread of 7.0 mm or less. Vascular space involvement, venous or lymphatic, does not affect classification.
T1a1	IA1	Measured stromal invasion 3.0 mm or less in depth and 7.0 mm or less in horizontal spread
T1a2	IA2	Measured stromal invasion more than 3.0 mm and not more than 5.0 mm with a horizontal spread 7.0 mm or less
T1b	IB	Clinically visible lesion confined to the cervix or microscopic lesion greater than T1a/IA2
T1b1	IB1	Clinically visible lesion 4.0 cm or less in greatest dimension
T1b2	IB2	Clinically visible lesion more than 4.0 cm in greatest dimension
T2	II	Cervical carcinoma invades beyond uterus but not to pelvic wall or to lower third of vagina
T2a	IIA	Tumor without parametrial invasion
T2a1	IIA1	Clinically visible lesion 4.0 cm or less in greatest dimension
T2a2	IIA2	Clinically visible lesion more than 4.0 cm in greatest dimension
T2b	IIB	Tumor with parametrial invasion
T3	III	Tumor extends to pelvic wall and/or involves lower third of vagina, and/or causes hydronephrosis or nonfunctioning kidney
T3a	IIIA	Tumor involves lower third of vagina, no extension to pelvic wall
T3b	IIIB	Tumor extends to pelvic wall and/or causes hydronephrosis or nonfunctioning kidney
T4	IVA	Tumor invades mucosa of bladder or rectum, and/or extends beyond true pelvis (bullous edema is not sufficient to classify a tumor as T4)

Regional lymph nodes (N)

TNM categories	FIGO stages	Definition
NX		Regional lymph nodes cannot be assessed
N0		No regional lymph node metastasis
N1	IIIB	Regional lymph node metastasis

Distant metastasis (M)			
**TNM categories**	**FIGO stages**	**Definition**	
M0		No distant metastasis	
M1	IVB	Distant metastasis (including peritoneal spread, involvement of supraclavicular, mediastinal, or paraaortic lymph nodes, lung, liver, or bone)	
**Anatomic stage/prognostic groups**			
Stage 0*	Tis	N0	M0
Stage I	T1	N0	M0
Stage IA	T1a	N0	M0
Stage IA1	T1a1	N0	M0
Stage IA2	T1a2	N0	M0
Stage IB	T1b	N0	M0
Stage IB1	T1b1	N0	M0
Stage IB2	T1b2	N0	M0
Stage II	T2	N0	M0
Stage IIA	T2a	N0	M0
Stage IIA1	T2a1	N0	M0
Stage IIA2	T2a2	N0	M0
Stage IIB	T2b	N0	M0
Stage III	T3	N0	M0
Stage IIIA	T3a	N0	M0
Stage IIIB	T3b	Any N	M0
	T1-3	N1	M0
Stage IVA	T4	Any N	M0
Stage IVB	Any T	Any N	M1

Note: cTNM is the clinical classification, pTNM is the pathologic classification.
* FIGO no longer includes Stage 0 (Tis).
▪ All macroscopically visible lesions-even with superficial invasion-are T1b/IB.

APPENDIX III
KARNOFSKY PERFORMANCE SCALE

100	Normal; no complaints; no evidence of disease
90	Able to carry on normal activity; minor signs or symptoms of disease
80	Normal activity with effort; some sign or symptoms of disease
70	Cares for self; unable to carry on normal activity or do active work
60	Requires occasional assistance, but is able to care for most personal needs
50	Requires considerable assistance and frequent medical care
40	Disabled; requires special care and assistance
30	Severely disabled; hospitalization is indicated, although death not imminent
20	Very sick; hospitalization necessary; active support treatment is necessary
10	Moribund; fatal processes progressing rapidly
0	Dead

APPENDIX IV
QUALITY OF LIFE ASSESSMENT (QOLA) FORM

EORTC QLQ-C30 (version 3)

We are interested in some things about you and your health. Please answer all of the questions yourself by circling the number that best applies to you. There are no "right" or "wrong" answers. The information that you provide will remain strictly confidential.

Please fill in your initials: |_|_|_|_|
Your birthdate (Day, Month, Year): |_|_|_|_|_|_|_|_|
Today's date (Day, Month, Year): 31 |_|_|_|_|_|_|_|_|

		Not at All	A Little	Quite a Bit	Very Much
1.	Do you have any trouble doing strenuous activities, like carrying a heavy shopping bag or a suitcase?	1	2	3	4
2.	Do you have any trouble taking a long walk?	1	2	3	4
3.	Do you have any trouble taking a short walk outside of the house?	1	2	3	4
4.	Do you need to stay in bed or a chair during the day?	1	2	3	4
5.	Do you need help with eating, dressing, washing yourself or using the toilet?	1	2	3	4

	**During the past week:**	Not at All	A Little	Quite a Bit	Very Much
6.	Were you limited in doing either your work or other daily activities?	1	2	3	4
7.	Were you limited in pursuing your hobbies or other leisure time activities?	1	2	3	4
8.	Were you short of breath?	1	2	3	4
9.	Have you had pain?	1	2	3	4
10.	Did you need to rest?	1	2	3	4
11.	Have you had trouble sleeping?	1	2	3	4
12.	Have you felt weak?	1	2	3	4
13.	Have you lacked appetite?	1	2	3	4
14.	Have you felt nauseated?	1	2	3	4
15.	Have you vomited?	1	2	3	4
16.	Have you been constipated?	1	2	3	4

Please go on to the next page

During the past week:	Not at All	A Little	Quite a Bit	Very Much
17. Have you had diarrhea?	1	2	3	4
18. Were you tired?	1	2	3	4
19. Did pain interfere with your daily activities?	1	2	3	4
20. Have you had difficulty in concentrating on things, like reading a newspaper or watching television?	1	2	3	4
21. Did you feel tense?	1	2	3	4
22. Did you worry?	1	2	3	4
23. Did you feel irritable?	1	2	3	4
24. Did you feel depressed?	1	2	3	4
25. Have you had difficulty remembering things?	1	2	3	4
26. Has your physical condition or medical treatment interfered with your family life?	1	2	3	4
27. Has your physical condition or medical treatment interfered with your social activities?	1	2	3	4
28. Has your physical condition or medical treatment caused you financial difficulties?	1	2	3	4

For the following questions please circle the number between 1 and 7 that best applies to you

29. How would you rate your overall health during the past week?

1 2 3 4 5 6 7

Very poor Excellent

30. How would you rate your overall quality of life during the past week?

1 2 3 4 5 6 7

Very poor Excellent

EORTC QLQ – CX24

Patients sometimes report that they have the following symptoms or problems. Please indicate the extent to which you have experienced these symptoms or problems, please answer by circling the number that best applies to you.

During the past week:	Not at all	A little	Quite a bit	Very much
31. Have you had cramps in your abdomen?	1	2	3	4
32. Have you had difficulty in controlling your bowels?	1	2	3	4
33. Have you had blood in your stools (motions)?	1	2	3	4
34. Did you pass water/urine frequently?	1	2	3	4
35. Have you had pain or a burning feeling when passing water/urinating?	1	2	3	4
36. Have you had leaking of urine?	1	2	3	4
37. Have you had difficulty emptying your bladder?	1	2	3	4
38. Have you had swelling in one or both legs?	1	2	3	4
39. Have you had pain in your lower back?	1	2	3	4
40. Have you had tingling or numbness in your hands or feet?	1	2	3	4
41. Have you had irritation or soreness in your vagina or vulva?	1	2	3	4
42. Have you had discharge from your vagina?	1	2	3	4
43. Have you had abnormal bleeding from your vagina?	1	2	3	4
44. Have you had hot flushes and/or sweats?	1	2	3	4
45. Have you felt physically less attractive as a result of your disease or treatment?	1	2	3	4
46. Have you felt less feminine as a result of your disease or treatment?	1	2	3	4
47. Have you felt dissatisfied with your body?	1	2	3	4

Please go on to the next page

During the past 4 weeks:	Not at all	A little	Quite a bit	Very much
48. Have you worried that sex would be painful?	1	2	3	4
49. Have you been sexually active?	1	2	3	4

Answer these questions only if you have been sexually active during the past 4 weeks:	Not at all	A little	Quite a bit	Very much
50. Has your vagina felt dry during sexual activity?	1	2	3	4
51. Has your vagina felt short?	1	2	3	4
52. Has your vagina felt tight?	1	2	3	4
53. Have you had pain during sexual intercourse or other sexual activity?	1	2	3	4
54. Was sexual activity enjoyable for you?	1	2	3	4

APPENDIX V
PHYSICS QUESTIONNAIRE FORM (PQF)

PHYSICS QUESTIONNAIRE FORM

Institution Name: ____ Date: ______________

Contact Information (name, address, phone, fax, email):

Physicist:

Radiation Oncologist:

Dosimetrist (if applicable):

Study Coordinator (if applicable):

TABLE 1. DELIVERY RESOURCES

List the treatment units you use. If units differ in the type of MLC or IGRT capabilities then list them separately. List all units that will be used on the protocol (add more lines to the Table if needed).

Unit #	Local unit identifier (name)	Vendor	Model	Photon energies used for IMRT	Number of additional identical units	MLC or other beam modulator (footnote 1)	Used for (IMRT, IGRT, both, or neither):	IMRT Method (see footnote 2)

Footnotes (enter a letter from the following list):

1. a. Varian 80 leaf; b. Varian Millennium 120 leaf; c. Elekta 80 leaf
   d. Elekta 80 leaf Beam Modulator; e. Tomotherapy Binary Collimator; f. NOMOS Binary Collimator
   g. Siemens 58 leaf; h. Siemens 82 leaf; i. 3D Line
   j. Radionics; k. BrainLAB 52 leaf; l. BrainLAB Tx 120 leaf
   m. physical compensators; n. CyberKnife using circular collimators
   o. Other: ____

2. p. SMLC; q. DMLC; r. Helical tomotherapy
   s. Serial tomotherapy; t. Other:____

TABLE 2. PLANNING RESOURCES

ID #	Vendor	Software Version	Calculation Algorithm (footnote 1)	Treatment units commissioned for this system (enter from Table 1)	Is system commissioned for heterogeneity corrections? (Enter YES or NO and see footnote 2)	Does the system transfer beams to a phantom for QA? (Enter YES or NO. If no, explain the technique you do use for IMRT QA in the space below)

Footnotes (enter a letter from the following list):

1. a. BrainLAB pencil beam b. Corvus pencil beam c. Helax pencil beam
   d. Helax collapsed cone e. Cadplan pencil beam f. Eclipse pencil beam
   g. Eclipse AAA h. PLUNC pencil beam i. MSKCC pencil beam
   j. Pinnacle fast convolve k. Pinnacle collapsed cone or adaptive convolution superposition l. XiO modified Clarkson or convolution
   m. XiO superposition or fast superposition n. Tomotherapy convolution superposition
   o. Other: ____

2. If you answered NO for the question about the system being commissioned for heterogeneity corrections, please explain? Identify each system using the # in the list:____

TABLE 3. IGRT RESOURCES

IGRT is defined here to include only procedures where an x-ray imaging technique is used in combination with some form of computer-assisted manual or automatic registration with the image information obtained during the patient's planning CT procedure. The standard use of MV EPID images as a visual comparison to DRRs does not fall under this definition. Also, the use of silver halide film radiographs alone is not accepted under this definition of IGRT.

Unit ID # (from Table 1)	Description of IGRT system (footnote 1)	How is image registration accomplished (footnote 2)?	How is the success of image registration verified (footnote 3)?	Do any of these units have robotic couches capable of correcting angular deviations (YES or NO)	How often do you check the position of the imaging system isocenter (footnote 4)?

Footnotes (enter a letter from the following list):

1. a. kV cone-beam (2D or 3D match) b. Dual kV imaging panels (e.g. ExacTrac, CyberKnife) c. Helical MV tomography
   d. MV cone beam e. In-room diagnostic CT scanner f. kV or MV stereoscopic images using EPID
   g. Other: ____

2. a. automatic registration b. manual click, drag, and rotate
   c. Other: ____

3. a. split screen b. spy glass c. color fade
   d. Other: ____

4. a. daily b. weekly c. monthly
   d. yearly e. not done
   f. Other: ____

QUESTIONNAIRE

1. How do you verify field positioning relative to the patient's anatomy (check all that apply)?
   ___________ Port Film
   ___________ Orthogonal Port Films
   ___________ Cone beam CT
   ___________ MVCT
   ___________ Other:____

2. Describe the method(s) used to conduct a check of the dose and monitor unit calculations generated by the 3DRTP system.
____

3. What record & verify system is used to monitor 3DCRT treatments (Manufacturer & Model):
____

4. How do you verify that the treatment unit delivers the planned dose for individual patients?
   A. Absolute Dose Point Measurement
      ___________ Ion Chamber (Chamber size:____________________)
      ___________ Diode
      ___________ TLD
      ___________ Radiographic Film
      ___________ Radiochromic Film
      ___________ Other: ____

   B. Relative Dose
      ___________ Isodose distribution with Radiographic Film
      ___________ Isodose distribution with Radiochromic Film
      ___________ Isodose distribution with Gel Dosimetry
      ___________ Other (specify # of axial, sagittal, and coronal planes): _________________________________

   C. Describe the type of phantom you use for QA:
      ___________ Anthropomorphic phantom (Vendor:_______________________________________)
      ___________ Geometric phantom
         Material:__________________________
         Shape:____________________________
         Size:_____________________________

   D. What agreement between planned and measured doses for individual patients is considered acceptable at your institution?
      Absolute dose in target volume (high dose region): _________________
      Absolute dose in critical normal tissue region:_____________________
      Absolute dose in low dose region:___________________________
      Relative dose in high dose gradient region:_______________________
      Relative dose in low dose gradient region:
         in high dose region (target):____________________________
         in low dose region:_________________________________

   E. Are your monitor unit calculations checked by an independent program?
      ___________ No
      ___________ Yes (Vendor: _______________________________)

5. In how many patients have you used IGRT for cervical cancer in the past 12 months?___________________________

6. With what frequency do you use IGRT?
   ______________ Each fraction
   ______________ First five fractions and once weekly thereafter
   ______________ Other:____

7. Do you perform a second IGRT study after the patient's position is adjusted?
   ______________ Yes
   ______________ No

8. What tolerance levels (in mm) are used for x, y, and z adjustments of the patient's position?_______________________

9. What are your rotational tolerances before repositioning the patient?____

10. If the system has a robotic couch, what are your tolerance levels for the rotational corrections? _______________________

11. Who approves the changes at the time of treatment?

_____________ Therapist
_____________ Radiation Oncologist
_____________ Physicist
_____________ Other:____

12. Is your treatment planning system (TPS) capable of using custom CT number to electron density tables or does your institution use the TPS vendor supplied numbers?

_____________ Custom CT number to electron density tables
_____________ TPS vendor supplied

13. If custom tables are used, how were the numbers generated?

_____________ Using measured data from CT
_____________ Other:____
